# 20-minute neighbourhoods, criticisms, conspiracy theories, and health: a critical discourse analysis

**DOI:** 10.1186/s12889-025-25416-y

**Published:** 2025-11-21

**Authors:** Melissa F. Legge, Elizabeth Inyang, Jonathan Stokes

**Affiliations:** School of Health and Wellbeing, College of Medical, Veterinary and Life Sciences, University of Glasgow, Clarice Pears Building 90 Byres Road, Glasgow, G12 8TB UK

**Keywords:** 15-minute city, 20-minute neighbourhood, Conspiracy, Conspiracy theories, Health, Equity

## Abstract

**Background:**

20-minute neighbourhoods (20MNs) and other proximity-based urban planning models are argued to improve communities, enhance population health, and reduce inequities. However, these links remain unevidenced. Conversely, there are science-driven criticisms that 20MNs could exacerbate health/social inequities requiring evaluation. These models have also resulted in conspiracy theories, believed to be linked to COVID-19 public health measures, which may hinder the implementation and evaluation of 20MNs and any expected health/inequity gains.

**Aim:**

The aim of this research is to better understand how public and media perceptions, criticisms, and conspiracy theories about 20MNs have evolved since the pandemic, how these link to health/social inequities, and how these connect to misinformation and disinformation of public health interest.

**Methods:**

A qualitative study design using thematic and critical discourse analysis (CDA). We systematically searched a selection of UK/US online newspapers from across the political spectrum for analysis. The timeframes of interest are the two 4-year periods before and after the pandemic was declared on March 11, 2020, to compare differences in discourse that may have resulted from the pandemic and its response.

**Results:**

Coverage of 20MNs increased significantly in the post-pandemic period. Through the analysis, we identified several themes, including restriction of movement/loss of privacy, social engineering, political affiliation, COVID-19 as an accelerator, worsening health/social inequities, classism, and liveability/resiliency.

**Conclusions:**

There is a significant political divide in 20MN discourse. 20MNs were linked to other conspiracy theories such as anti-vaccine beliefs and climate change denial. Framing of these interventions may be a contributing factor.

**Supplementary Information:**

The online version contains supplementary material available at 10.1186/s12889-025-25416-y.

## Introduction

The 15-Minute-City (15MC) and 20-Minute-Neighbourhood (20MN) are urban planning models gaining policy traction. They aim to place essential services such as schools, grocery stores, and healthcare within a 15–20-minute walk, bike ride, or transit trip from any point in a community [1]. These models build on earlier proximity-based concepts like walkable, liveable, garden, and compact cities, some dating back to the 1800s [2]. In more recent iterations, their goal has expanded into a multi-pronged focus, including health outcomes and equity, by emphasizing sustainability and accessibility through reduced car dependence, active transportation, and climate change mitigation [3].

While the general principles of 20MNs remain largely unchanged since their introduction nearly a decade ago, recent public protests and backlash signal a shift in discourse that is not yet well understood [4, 5]. Criticisms range from equity concerns, all the way to those labelled as conspiracy theories [6]. Understanding this change in discourse is vital if any of the proposed health (equity) benefits are to be realised. This research highlights key knowledge gaps, examines the potential influence of COVID-19 on public discourse, and analyses media narratives using critical discourse analysis (CDA).

## Background/literature review

### 20MN health claims

20MNs are commonly cited as improving health and reducing inequities by improving place-based factors that influence the social determinants of health (SDOH) [4]. 20MNs might support access to healthcare, healthy food, employment, and greenspaces [2]. Reducing commutes is thought to improve quality of life by freeing time for social, creative, or recreational pursuits [4]. 20MNs may also encourage active transportation, which is linked to a lower risk of non-communicable diseases [7]. Additionally, they are promoted as a sustainable alternative to traditional urban design for longer-term health pathways, reducing carbon emissions through traffic reduction, infrastructure improvements, decreased urban sprawl, and mixed-use zoning [4].

### Science-driven criticisms

Despite promising health claims, increased policy development and early implementation have prompted empirical critiques. The link between 20MNs and health remains debated. In many communities, achieving 20MN status would require substantial redevelopment, raising concerns about gentrification, displacement, and social exclusion [2, 8, 9]. Some researchers argue that 20MNs may worsen health/social inequities, while others suggest they could improve liveability in socioeconomically deprived neighbourhoods [9, 10]. Neither position has been proven empirically, since the implementation tends to be at the early or only planning stages [2].

### Misinformation, disinformation, and conspiracy theories

In addition to evidence-based criticisms, some groups and individuals view 20MNs as a threat to personal rights and freedoms. Although planning for more accessible communities is outwardly innocuous, claims have surfaced in news, social media, and political debates that 20MNs are a pretext to impose “climate lockdowns” [11]. Critics allege that 20MNs aim to confine the public to “districts” under surveillance [12]. Existing 20MN policies have not endorsed lockdowns or confinement and, as with the proposed benefits, there is no empirical evidence supporting these claims [13].

Researchers and planners should aim to address, rather than dismiss these claims, as they may reflect underlying societal fears and searches for meaning amid uncertainty [14, 15]. Mis/disinformation can harm public health, worsen inequities, and erode trust in institutions [16]. Failing to address 20MN conspiracy theories with evidence may impede implementation, evaluation, and potential benefits of these interventions [17]. However, there is academic debate regarding how to distinguish conspiracy theories from critical theory, as these often share superficial similarities [18]. Therefore, it may be challenging to differentiate where criticism becomes conspiracy. Understanding key sociopolitical themes of this discourse may better equip those involved in public health and urban planning to identify and address conspiracy theories, understand any underlying legitimate concerns, and better communicate potential benefits. The following sections define key terms: misinformation, disinformation, and conspiracy theories.

#### Misinformation and disinformation

Misinformation and disinformation both refer to misleading or false information but they are distinguished by intent. Misinformation is generally not known to be false, whereas disinformation is intentionally inaccurate and shared to deceive or manipulate [19].

#### What constitutes a conspiracy theory?

Douglas et al. [20] define a conspiracy theory as “a belief that two or more actors have coordinated in secret to achieve an outcome and that their conspiracy is of public interest but not public knowledge” (pg. 282). Douglas identifies five key elements of conspiracy theories:

1. They dispute mainstream perceptions of events.
2. They evoke nefarious or unacceptable actions.
3. Blame is placed on individuals, groups, or organizations, rather than systemic factors.
4. Collectively, they pose epistemic risk when compared to other beliefs.
5. They represent shared belief systems with social objectives that can potentially shape social realities [20].

### Potential relation to COVID-19 and knowledge gaps

Recent public health responses to COVID-19 are another policy area to have sparked controversy, criticisms, and conspiracy theories [21–23]. In the early pandemic, limited knowledge of SARS-COV-2 and rapid information sharing via news and social media created an ideal environment for conspiracy theories [24]. The pandemic may also be spilling over to hesitancy towards other public health measures, including routine immunizations [25]. Few studies have systematically examined how 20MN criticisms/conspiracy theories have evolved in this context; most existing work consists of commentaries or editorials. Research into public and media perceptions of 20MNs remains limited. Thus, critically analysing 20MN media discourse is essential to inform public health, urban planning, and policy. COVID-19 is one of several factors potentially shaping this discourse. A timeline of relevant events appears in Appendix 1.

## Research questions (RQs)

1. How have public/media perceptions of 20MNs changed since the COVID-19 pandemic?
2. How is health/social equity represented in the pre- and post- pandemic 20MN public/media discourse?
3. Is the content of 20MN criticisms/conspiracy theories linked to other prevalent misinformation/disinformation of public health interest, such as COVID-19 conspiracies, anti-vaccine beliefs, and climate change denial?

## Methods

Methods were adapted from Comer and Noones’ (2024) [26]. Written and spoken language, particularly online, is a primary channel through which misinformation, disinformation, and conspiracy theories spread [15]. CDA emphasizes language as a means of exerting power and creating, upholding, or challenging power dynamics [27–29]. 20MN criticisms/conspiracy theories often reflect distrust in institutional power granted to planners by government. CDA was used to examine how the sociopolitical contexts of 20MN discourse may reinforce or challenge inequities and influence societal beliefs and norms [30]. We followed Mullet’s [31] seven-step CDA framework (see Table 1): select a discourse; locate/prepare data; explore textual background; identify codes and themes; analyse external/internal textual relations; and interpret the data [31].

**Table 1:** Analytic process

Stage of Analysis		Description	Example
1	Select the discourse	*Select a discourse related to injustice or inequality in society.*	• 20MN discourse, criticisms, and conspiracy theories in online news sources pre- and post-COVID-19 pandemic
2	Locate and Prepare Data Sources	*Select data sources (texts) and prepare the data for analysis.*	• Articles from selected sources meeting inclusion criteria • Import into NVivo15
3	Explore the background of each text	*Examine the social and historical context and producers of the texts.*	• Social/historical contexts of 20MNs and similar frameworks (intro/background) • Subgroups that may be impacted by 20MN policies based on historic factors (methods) • Political leanings of each source (methods) • Ownership and funding of each publication (results)
4	Code texts and identify overarching themes	*Identify the major themes and subthemes using choice of qualitative coding methods.*	• Inductive/deductive coding in NVivo15 (results) • Thematic analysis (results)
5	Analyse the external relations in the texts (interdiscursivity)	*Examine social relations that control the production of the text; in addition*,* examine the reciprocal relations (how the texts affect social practices and structures). How do social practices inform the arguments in the text? How does the text in turn influence social practices?*	• Social relations – political beliefs, social norms • Reciprocal relations – how people engage with 20MN policies • Social practices – car use/dependence, walking, cycling, use of services (results/discussion)
6	Analyse the internal relations in the texts	*Examine the language for indications of the aims of the texts (what the texts set out to accomplish)*,* representations (e.g.*,* representations of social context*,* events*,* and actors)*,* and the speaker’s positionality.*	• Subject positioning of 20MNs, protests, conspiracy theories, advocates i.e., “quacks”, “pro-car protestors” • What the articles aim to accomplish (results/discussion)
7	Interpret the Data	Interpret the meanings of the major themes, external relations, and internal relations identified in stages 4, 5, and 6.	• Report findings and relate these to PROGRESS-Plus, and wider literature (results/discussion)

### Step 1: selecting the discourse

The selected discourse was representations of 20MNs in online news sources from the United Kingdom (UK) and United States (US), where 20MN frameworks have been implemented to varying degrees. In this study, we follow Wodak’s [32] definition of “discourse” as structured forms of knowledge about social practices that both shape and are shaped by social and political contexts. All selected sources engage with the 20MN concept, though their coverage reflects differing ideological and political perspectives. For this analysis, we treat these representations as a singular discourse, acknowledging the ideological differences between sources.

Political bias can significantly alter media tone and content, and belief in conspiracies is often linked to political extremism [33, 34]. To account for this, one mainstream, one far-left, and one far-right news publication were selected from each country. Political leanings were determined using Media Bias/Fact Check (MBFC), which applies a rigorous methodology to classify media on a political spectrum from “extreme left” to “extreme right”, and provides information on factual accuracy, credibility, and user traffic [35]. Selected UK publications were *The Times of London & The Sunday Times (TOL)* (mainstream), *Novara Media* (far-left), and *Spiked Magazine* (far-right); the US publications selected were *The New York Times (NYT)* (mainstream), *Counterpunch* (far-left), and *The Federalist* (far-right) (Appendices 3 and 4 detail each UK and US publication’s political leaning, factual reporting/credibility ratings, and traffic levels as rated by MBFC).

### Step 2: locating and preparing data sources

#### Search strategy

Pre- and post-pandemic were the timeframes of interest for this research. The pre-pandemic period was four years prior to the pandemic declaration on March 11, 2020 (March 10, 2016 – March 10, 2020) [36]. For equal comparison, the post-pandemic period was the four-year period following (March 11, 2020 – March 11, 2024), encompassing both the COVID-19 pandemic and the period after The World Health Organization (WHO) declared the end of the COVID-19 global health emergency (May 5, 2023). For conciseness, this will be referred to as the “post-pandemic” period throughout.

Searches were conducted directly on each publication’s website. Several terms were searched; search terms (see Appendix 2) were entered individually using quotation marks for exact matches. Articles outside the specified dates and duplicates were manually removed. Boolean operators (e.g., “AND”, “OR”, “NOT”) were not used, as not all websites supported them. Results were compiled in an adapted PRISMA flow diagram [37]. Articles were excluded if they were the wrong type or inaccessible; specific exclusion reasons are listed below.

#### Inclusion and exclusion criteria

All articles were manually reviewed. Those with only passing mentions of 20MNs were excluded as “irrelevant”. Information on all articles meeting inclusion criteria, including publication, article names, hyperlinks, publication dates, political orientation, article stance on 20MNs (supportive, mixed, against, etc.), and potential conflicts of interest, is provided in Appendix 18.

#### Inclusion criteria

- Published in a selected source.
- Contains search term in headline or article body.
- Free access or behind a paywall.
- 20MN or related concepts are a primary focus.

#### Exclusion criteria

- Newsletters.
- Travel guides.
- Real estate/property buying guides.
- Broken links.
- Only brief mentions of 20MN concepts.
- Premium or extra content requiring full subscription.

### Step 3: background of each text

Each publication’s website and MBFC were reviewed, and we conducted supplementary Google searches to gather information on ownership and funding, offering insight into potential conflicts of interest. Transparent revenue streams such as subscriptions, advertisements, premium content, and paywalls are also noted in table format in Appendix 5.

### Step 4: coding and identifying themes

Coding was completed using NVivo15, following Allsop et al.’s guide [38]. Codes were selected a priori based on the research questions (deductive, see step 7) or identified from reading articles (inductive) [39]. Similar codes were then sorted into themes.

#### Thematic analysis

To systematically identify patterns and themes in the data, thematic analysis (TA) was applied as part of Step 4 in Mullet’s (2018) CDA framework [31]. We utilized Naeem et al.’s (2023) step-by-step TA process to enhance rigour and transparency [40]. This integration ensures that TA and CDA are complementary, rather than separate analytic approaches.

### Step 5: analysing external relations

To determine external relations informing each text, we examined social relations, reciprocal relations, and social practices [31]. Appendix 6 outlines external relations examined within each text.

### Step 6: analysing internal relations

To determine internal relations informing each text, we examined apparent aims, representations, and positionalities [31]. Additionally, following Gordon [41] and Van Dijk’s [42] guidance, we examined the subject positioning, discursive framing, and mental models in the included articles. Appendix 7 outlines internal relations examined within each text.

### Step 7: interpret the data (analysis)

#### Change in content (RQ1)

RQ1 aims to determine if the pandemic contributed to increased 20MN criticisms/conspiracy theories. A confounding factor is that COVID-19 may also have contributed to increased 20MN policy implementations, leading to criticisms. To test these hypotheses, we applied the CDA framework in comparing content of 20MN media discourse pre- and post-pandemic. Deductive codes relating to RQ1 include *COVID-19 accelerated 20MN conspiracy theories* and *COVID-19 accelerated* [the implementation of] *20MNs*.

#### Linking the DA to equity (RQ 2)

Deductive codes relating to RQ2 include *improve health*,* worsen inequities*, and *reduce inequities*, as we hypothesised these conflicting perspectives would appear in the discourse. *Worsen inequities* was created as a parent code, with child codes reflecting the PROGRESS-Plus framework and groups at higher risk for health/social inequities [11, 43]. Incorporating PROGRESS-Plus ensured that differences related to place of residence, race/ethnicity, occupation, gender, religion, education, socioeconomic status (SES), social capital, and other relevant factors were systematically considered during coding, allowing for a transparent and equity-informed analysis [11].

#### Linking the DA to misinformation/disinformation (RQ3)

Deductive codes relating to RQ3 reflect prevalent health-related conspiracy theories that we suspected may appear in media, such as *anti-vaccine* and *climate change denial*. Links to any other, non-expected conspiracy theories were coded inductively.

### Ethics

Ethics approval was not required, in accordance with the guidelines of the University of Glasgow College of Medical, Veterinary and Life Sciences Ethics Committee, as this research was carried out as secondary analysis of publicly available publications [44]. News media is published with no expectation of confidentiality or privacy, so informed consent from individual authors was not required [45]. Although a formal ethics approval process was not required for this review, the authors carefully considered the ethical dimensions throughout the research process, engaging in detailed discussions about responsible presentation of findings.

## Results

### Search results

**Fig. 1:**
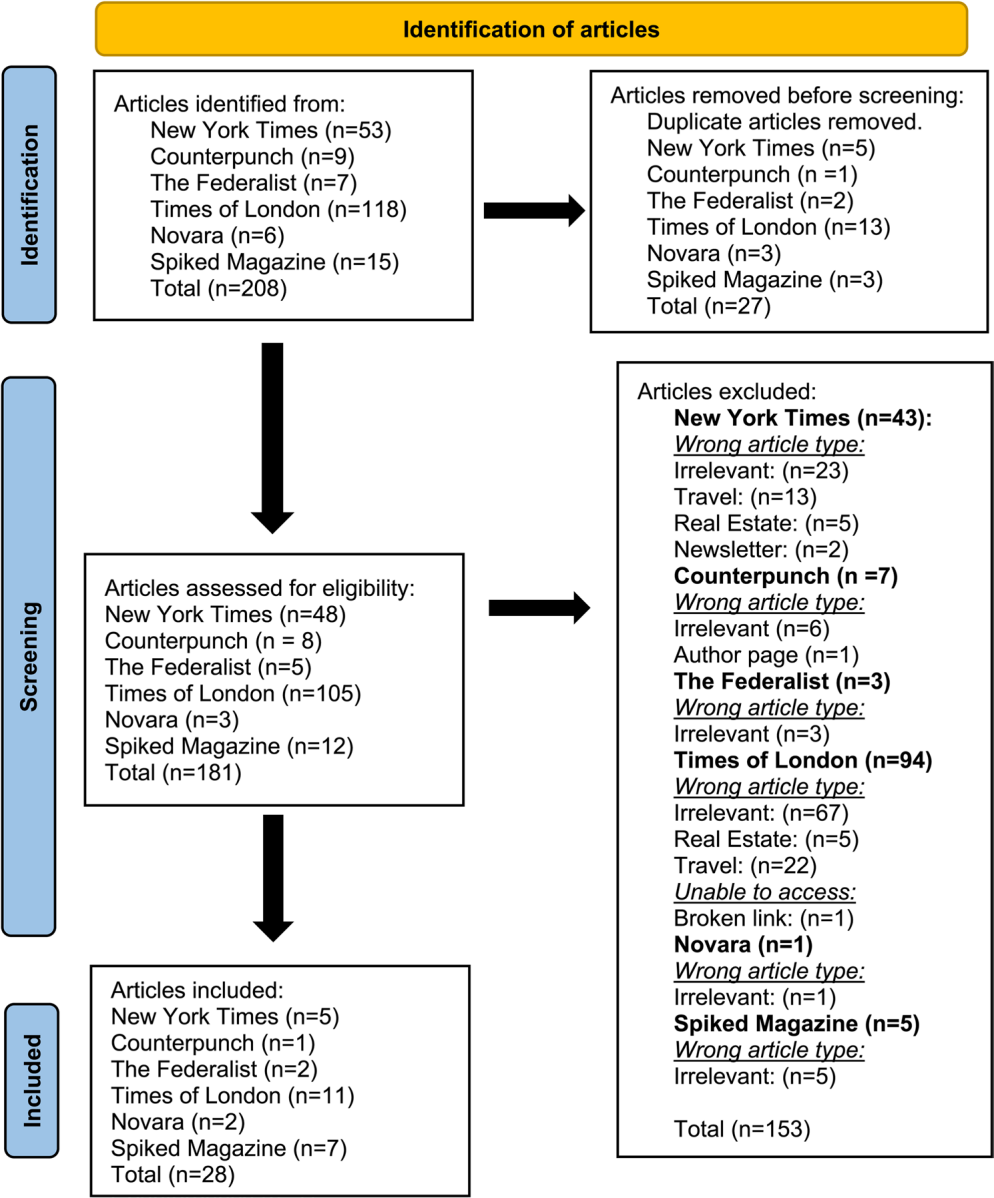
PRISMA diagram

208 initial search results were returned with 27 duplicates removed. 181 articles were assessed for eligibility; 153 articles were excluded. 28 articles met inclusion criteria (see Fig. 1). Appendices 8–9 illustrate search results and ownership and funding information.

### Codes/themes

#### Codes

Several inductive [34] and deductive [7] codes were identified, reflecting a range of perceptions towards 20MNs. Code examples and the full code book can be found in Appendices 10–11 with deductive codes highlighted.

#### Themes

Themes identified under RQ1 were *restriction of movement/loss of privacy*,* social engineering*,* political affiliation*,* and COVID-19 as an accelerator.* Themes identified under RQ2 were *worsening inequities*,* classism*,* resiliency*, and *liveability.* Themes identified under RQ3 were *anti-vaccine* and *the great reset/climate change denial;* these are each discussed in more detail below.

### Evolution of 20MN discourse (RQ1)

Pre-pandemic, 20MNs were not a central focus of any published article in the selected sources. Minimal critical reporting appeared in the first half of the post-pandemic period. In a 2020 mainstream article, motorists were represented as “angered” by Paris’s implementation of 20MNs [46]. In 2021, another positioned 20MNs as “greenwashing” [47].

Conspiracy-related coverage began in early 2023, aligning with anti-20MN protests in Oxford, UK and critical remarks from conservative politicians about 15MCs. Key post-pandemic themes included *restriction of movement/loss of privacy*,* social engineering*,* political affiliation*, and *COVID-19 as an accelerator.*

#### Restriction of movement/loss of privacy

*Restriction of movement* captures “climate lockdown” narratives and beliefs that 20MNs are “anti-car”, will result in confinement, and resemble ghettos/concentration camps [48–50]. *Loss of privacy* is a subtheme, involving claims that compliance will be enforced through invasive surveillance [48]. Depending on the source these were framed either as possible/inevitable outcomes or as outlandish conspiracy theories.

A far-right article titled “*Climate Psychos Want To Abolish Freedom Of Movement*” claimed:

*“These [15MCs] will maintain watch and control over the populace’s movement via sophisticated surveillance technology that is already being installed across the world and promoted in the West by intelligence groups like the CIA*,* FBI*,* and Mossad.”* [48].

Here, climate activists are represented as “psychos”, implying mental instability. The article positions 15MCs alongside clandestine organizations, encouraging readers to draw a false equivalency between these. This framing may reinforce existing conspiratorial thinking. The claim lacks credible evidence and links to another media outlet MBFC classifies as a pseudoscience and conspiracy website, suggesting an intentional effort to radicalize readers through a chain of increasingly alarming narratives [51].

Another far-right article “*The ‘[15MC]’ is not a conspiracy theory*” takes the positionality of concerned citizens being unfairly dismissed as “conspiracy theorists” [52]. It suggests leaving 15MCs could “potentially [lead] to fines”, a claim inferred from Oxford’s Low Traffic Neighbourhood (LTN) plan, not 15MC policies directly [52]. This misrepresentation, whether from neglect or intent, could cause heightened emotional responses and negative engagement with 20MN policies. Both far-right publications framed 20MNs as a “war on cars”, a metaphor evoking threat and urgency, while ignoring pedestrian and cyclist rights – demonstrating selective attention to motorist concerns [53].

Conversely, one mainstream article “*The new Tory conspiracy theory? A 15-minute stroll is ‘sinister’*” mocks these beliefs, using sarcasm and humour to discredit critics [49]. It portrays conservatives as gullible but offers no fact-checking or counterevidence [49]. This framing may reinforce existing beliefs of persecution in conspiracy thinkers, while others may use this to dismiss 20MN criticisms without critical evaluation.

#### Social engineering

*Social engineering* refers to how 20MNs influence urban behaviours and social structures. This includes fostering community connection, encouraging active transportation, and discouraging driving. Media representations of social engineering varied significantly. A far-right source framed it as coercive and manipulative, positioning climate activists as “fanatics” to discredit climate action and policy [50]. A far-left source frames social engineering as common and benign, ironically noting that current driving behaviours can also be viewed as social engineering [54]. A mainstream article, while initially sceptical, ultimately positioned the rationale as “disarming”, portraying planners as lacking substantial decision-making power [55].

These framings illustrate a spectrum of acceptance: far-left sources being most supportive, mainstream cautiously receptive, and far-right most critical. Examining the internal relations informing these texts, each publication aims to connect with their target audience through their discourse.

#### Political affiliation

Many publications noted that stances on 20MNs are shaped by political beliefs. Far-left and mainstream sources were critical of 20MN conspiracy theories and the far-right [49, 54, 56]. The far-right claimed their concerns were not inherently political, instead framing several climate action policies as government overreach [48, 57, 58]. A common feature across the political spectrum was a failure to engage productively with opposing views. In many cases, authors criticized conflicting beliefs without providing credible evidence or well-structured arguments to counter these. These results highlight significant political polarisation in the 20MN discourse, though the motives and rationale behind opposing stances differed by political affiliation.

#### COVID-19 as an accelerator

This theme included beliefs that the pandemic accelerated both the implementation of and criticisms/conspiracy theories about 20MNs. There was apparent consensus across the political spectrum that COVID-19 accelerated 20MNs, though the reasons and perceived impacts differed. For example, one mainstream source wrote:

*“What accelerated [20MNs] implementation by cities round the world*,* however*,* was Covid. Many found they could work at home quite easily and*,* freed from hellish commutes*,* fell back in love with their local environments”* [55].

This frames long commutes and rigid working arrangements as problems, and 20MNs as a solution. Conversely, a far-right source presented a different link between 20MNs and COVID-19:

*“- it was only after [COVID-19] lockdowns that the previously unthinkable idea of confining people to their local areas for the greater good was able to gain currency.*” [58].

This quote frames 20MNs as a continuation of problematic COVID lockdowns. These statements reflect differing aims, to increase or decrease public support for 20MNs, respectively.

Mainstream and far-left publications frequently positioned COVID-19 as accelerating 20MN conspiracies [59–61]. This theme was less prominent in far-right sources, which often rejected the term “conspiracy theory”. However, given the broad consensus that COVID-19 accelerated 20MNs, this may partly explain the increase in far-right criticisms post-pandemic. COVID-19 themes also appeared frequently in articles critical of 20MNs [48, 52, 58, 62].

### Health equity (RQ2)

Several codes linked to PROGRESS-Plus emerged in the discourse. Identified themes include *worsening inequities*, *classism*, *liveability*, and *resiliency*.

#### Worsening inequities

This theme reflects assertions that 20MNs exacerbate health/social inequities, disproportionately impacting equity-denied groups, including people with disabilities, women, and those of low socioeconomic status (SES).

##### Accessibility

Far-right media expressed concerns about the potential impacts of 20MNs on people with disabilities. One article titled “*The Classist War on the Car*”, stated:

*“The adjective ‘liveable’ is always thrown around by the eco-elites who hate cars. But who*,* exactly*,* will find cities ‘liveable’ under today’s anti-car hysteria? Not disabled people*,* that’s for sure.”* [63].

Pedestrian-centred planning is represented as “anti-car hysteria”, without addressing potential benefits. The author overlooks how existing infrastructure may already disadvantage people with disabilities, reflecting status-quo bias.

##### Gender

The same article claims 20MNs would harm women, particularly “housewives” and “mums”, speculating that 20MNs will make driving, shopping, and daily life more difficult for them [63]. This presents a narrow view of women’s roles and needs, relying on traditionalist conversative social relations, viewing women primarily as mothers and homemakers. The article further claims that 20MNs will burden “workmen”, because they “have tools”, reflecting a gender-biased view of who participates in the workforce [63]. Again, the author displays status-quo bias by overlooking how 20MNs might improve women’s lives.

##### SES

Far-right media linked 20MNs to fines from low-emissions zones and LTNs, framing them as discriminatory. However, this argument excludes the most socioeconomically deprived, who often rely on public or active transportation. The article stated:

*“In the warped narrative of the motorphobes*,* car-owners are part of ‘the privileged.’ This is nonsense. It is not prohibitively expensive to own a car in the 2020s.”* [63].

This is a broad generalization of who owns a car. For many, even necessities like food and housing are prohibitively expensive. The author fails to consider how current urban design perpetuates socioeconomic inequities. Inflammatory language, such as “eco-elite”, “anti-car”, and “motorphobe,” is used to elicit strong emotions. While inequity is presented as an argument against 20MNs, the article offers no concrete rationale, evidence, or solutions.

A few mainstream sources noted that 20MNs might exacerbate socioeconomic divides, presenting these concerns with nuance and evidence [64, 65]. No far-left articles raised this issue.

#### Classism

*Classism* reflects the view that 20MNs are anti-working class and benefit only the wealthy elite. These themes appeared primarily in far-right publications, one source wrote about individuals and organisations promoting 20MNs and other climate-conscious initiatives:

*“—consider how the climate activists that head C40 Cities*,* the [UN]*,* and the World Economic Forum (WEF) continue to galavant across the globe on private jets amid this supposed ‘climate crisis.’ They don’t want to give up air travel and SUVs — they just want to price out regular people from driving cars and flying commercially.”* [48].

This quote elicits hypocrisy and cognitive dissonance. The author draws on climate change denial/scepticism social relations by using quotation marks and the word “supposed” to describe the climate crisis.

The far-right also represented 20MNs as driving up living costs and economically punishing the middle class [48, 63]. Analysing the social relations and practices informing these texts, this discourse appears to target a middle-class, suburban, and car-dependent audience. This framing aims to resonate with and provoke a sense of injustice in this population.

#### Liveability and resiliency

The idea that 20MNs improve liveability and community resilience to COVID-19, climate change, and other public health emergencies was common in mainstream and far-left media. These concepts are influenced by PROGRESS-Plus factors such as place of residence and SES [11]. A mainstream article on Scottish 20MNs positions residents as fortunate to live near essential services [66]. While noting evidence-based concerns, the author balances benefits and critiques by citing credible sources and including voices of researchers and residents.

Some articles claimed 20MNs suffered fewer economic losses during the pandemic. A mainstream article titled “*How One San Francisco Street Survived the Pandemic”* frames 20MNs positively, suggesting they can support pandemic recovery [67]. It references social practices, such as shopping, socializing, and walking safely within a community [67]. However, attributing a neighbourhood’s success solely to being a 20MN is a broad generalization. The article overlooks other contributing factors, such as high SES, offering a biased representation. This may reinforce *classism* narratives and beliefs that 20MNs are only feasible in wealthy or privileged areas.

Resiliency to climate change also emerged as a theme. A far-left source referenced 15MCs in a listicle titled “*This is How Britain Can Actually Prepare for Extreme Weather”*, framing them as a solution to transportation disruptions due to natural disasters [68]. The article positions 20MNs alongside measures like reducing consumption as paths to climate change resiliency. Drawing parallels between the pandemic and climate change, the author frames both as emergencies of equal measure. This positionality may resonate with climate activists but could result in further opposition from those who view 20MNs as veiled lockdowns.

### Links to misinformation of public health interest (RQ3)

20MN criticisms were both framed as and compared to other conspiracies in mainstream and far-left media. Conversely, far-right sources consistently rejected the “conspiracy theorist” label.

#### Anti-vaccine

Far-left and mainstream publications linked 20MN criticisms/conspiracy theories to anti-vaccine beliefs [59, 60, 69]. While far-right sources did not explicitly mention vaccine opposition in their 20MN reporting, they often portrayed COVID-19 restrictions as an attack on personal liberties, implicitly including vaccine mandates [58, 70].

#### The great reset/climate change denial

20MNs were also associated with criticisms of the World Economic Forum (WEF), which endorsed 15MCs in its “Great Reset” pandemic recovery plan. This became a prominent conspiracy theory, believed to be linked to climate change denial [71]. One far-right source represented the WEF and C40-cities as using climate policy to exert control over the public [48], while another rejected this connection [50, 52]. Despite differing views on the WEF, both far-right sources expressed scepticism or denial of climate science:

*“Question any aspect of the climate-alarmist agenda*,* including the harebrained claim that billions will soon die in a fiery apocalypse of man’s making*,* and you’ll be branded with that D-word. It marks you out as unfit for public life.”* [50].

This quote portrays disdain for the term “climate change denier”. However, describing climate action as “alarmism” indicates scepticism, if not outright rejection, of climate science.

## Discussion

### Principal findings

Coverage of 20MNs has increased significantly since the pandemic, with clear partisan divides. Engagement between opposing perspectives was limited. Across the political spectrum, there was consensus that COVID-19 accelerated 20MN policy implementation. Whether different criticisms of 20MNs constituted conspiracy theories was debated. Far-right media rejected the “conspiracy theorist” label, framing 20MNs as threats to freedom and liberty, yet offered no credible evidence and often shared disinformation. These sources argued 20MNs could worsen inequities but displayed attentional, gender, and status-quo biases. On the other hand, mainstream and far-left publications dismissed these concerns as conspiracy theories. They highlighted potential 20MN benefits but often failed to address science-driven critiques. 20MN criticisms/conspiracy theories were also linked with other prominent public health controversies, including anti-vaccine beliefs and climate change denial.

### Strengths and limitations

This research has several strengths and limitations to consider when interpreting the findings. A key strength of CDA is its ability to provide holistic insights into the social constructs and perceptions shaping discourse [72]. However, CDA is interpretative and limited to researcher subjectivity [73]. All sources were screened, coded, and analysed by a single reviewer, which may introduce bias.

Using media as the primary data source offered a rich, varied sample of 20MN perceptions. Systematically searching publications across the political spectrum allowed for the capture of multiple perspectives and competing narratives. We applied evidence-based, peer-reviewed methodology throughout, such as Mullet’s framework [31], Allsop’s guide [38], and PROGRESS-Plus [11]. However, while we explicitly set out to use the PROGRESS-Plus framework in our methods, the actual discussion of relevant subgroups within the sources was limited, limiting the depth of analysis in terms of equity.

A limitation of this study is that no articles meeting the inclusion criteria were identified in the pre-pandemic period (March 10, 2016 *—* March 10, 2020). As a result, the analysis primarily reflects discourse occurring during and after the onset of the COVID-19 pandemic. This restricted the ability to directly compare pre- and post-pandemic representations of 20MNs and may limit generalizability to earlier periods.

Another key limitation is the political leaning of the “mainstream” publications. MBFC classifies *NYT* as left-centred and *TOL* as right-centred. Including a broader range of political perspectives may capture additional nuance, but identifying truly centrist sources with appropriate search functionalities was not feasible.

Finally, it was also beyond the scope to include articles where search terms only appeared briefly, social media discourse, additional UK/US publications, or sources from additional countries. Given the small sample size, these results may not be generalizable or replicable across other media or regions.

### Relation to other literature

#### Change in content

##### Restriction of movement

Restriction of movement, particularly by car, was a key theme in far-right media. This has been noted in prior literature and is thought to stem from perceived personal burdens and disruptions to travel routines caused by 20MNs [12]. The primary goal of 20MNs is not to restrict movement or burden motorists, but to create cities where alternative transportation is more viable and appealing, naturally reducing car use [4].

Cities with cycling infrastructure report higher cycling rates and improved safety for all road users, supporting the idea that more people choose active transportation when it is safe and feasible [74]. A counterpoint to the “war on cars” narrative is that opposing 20MNs could be viewed as restricting freedom of choice for those who would prefer to walk, cycle, or take public transit.

However, with increasing single-use developments and urban sprawl, car dependence has become the norm [75]. For many, cars represent freedom, individualism, and independence [76]. Early automakers lobbied governments and developed psychological marketing to embed this message into consumers’ minds. This may be linked to why perceived attacks on cars are often seen as attacks on liberty [75]. This CDA reinforces those findings and shows that even interventions not directly targeting car use may still be viewed through this lens. What remains unclear is which framing strategies may increase acceptability or best counter 20MN misinformation, disinformation, and conspiracy theories.

##### Social engineering

Across the political spectrum, social engineering was a recurrent theme in the 20MN discourse. Concerns that 20MNs may unintentionally worsen inequities through social engineering remain an underexplored gap in the literature [2, 9]. However, social engineering is not inherently negative and is commonly used in urban design. For example, garbage cans are installed to prevent littering and cross walks are placed so that people do not walk into traffic [77, 78]. These features promote safety, cleanliness, and efficiency. Echoing Marquet et al. [12], these findings suggest that successful 20MN implementation requires planners to acknowledge and address fears of social engineering and perceived personal sacrifices.

##### Political divide

The results indicate significant political division in 20MN discourse. Liberals and conservatives may exhibit morality bias, assuming opponents lack values, which leads to dehumanisation and a refusal to engage [79]. This bias was evident in far-right media portraying climate activists as supporting ghettoisation and unconstitutional movement restrictions [48, 58]. The mainstream and far-left media dismissed 20MN criticisms as far-right conspiracy theories [49, 54, 56]. Politically divisive coverage can increase negative perceptions of opposing parties [80]. These findings suggest that 20MN discourse may deepen partisan divides. Addressing morality bias may be a useful strategy to improve public acceptability and to reduce polarisation around 20MN planning. Engaging with the points made by the other side constructively might also aid future discourse.

#### Health/social equity

##### Inequity

20MNs are believed to reduce health/social inequities by improving access to local health-promoting services [4]. However, there is currently a lack of high-quality evidence supporting these claims [2]. For example, McGowan et al. [81] found in their systematic review of place-based interventions that improving built environments such as housing, transit, walking/cycling routes, and food access may increase physical activity and improve health. However, included studies did not report on PROGRESS + factors, limiting equity assessments [81].

Themes from this CDA underscore the need for more research into 20MNs impacts on inequities. This evidence gap has allowed misinformation and conspiracy theories to thrive [82]. Far-right media emphasised status-quo bias and the potential for 20MNs to worsen inequities. Future proposals should highlight how current urban design contributes to inequity and clarify that scaled implementation is essential for evaluating real-world impacts. Transparent communication and scenario modelling may help anticipate unintended adverse effects and inform more equitable planning [2].

##### Accessibility

Accessibility concerns were raised under the theme of *worsening inequity*. Far-right media claimed that 20MNs would harm people with disabilities. However, others argue that cities already prioritise cars, leading to congestion and limiting pedestrian space, which contributes to inaccessibility [83]. Hatzakis et al. [83] advocate for rethinking land use and ensuring stakeholder engagement to address disability needs. While no single intervention can fully solve inaccessibility, strong evidence supports the need for intervention [84–86]. Overstating current accessibility and exaggerating potential 20MN pitfalls without evidence reflects a potential lack of genuine concern for disability rights.

##### Gender

One far-right source claimed 20MNs would exacerbate gender inequities, suggesting the status-quo is preferable. This argument is unsupported by evidence, as pedestrian-friendly, socially connected neighbourhoods with quality transportation and infrastructure can enhance women’s safety and quality of life [87]. Kalms and Kalms [87] cite gender-bias and male violence in cities, emphasizing that creating women-centred cities requires “a participatory feminist framework” in urban design and direct input from women.

##### SES/classism

Concerns that 20MNs will worsen socioeconomic divides were common in far-right media and occasionally noted in mainstream sources. While this analysis adds little to the evidence base of how 20MNs may affect wealth disparities, it offers insights into how these narratives are framed. Far-right sources emphasised rising costs for the middle and working-class, reflecting broader fears of perceived personal sacrifices [12]. Mainstream articles mentioned segregation and marginalisation, though often as abstract or hypothetical concerns.

Notably, gentrification was rarely discussed in media, despite this being a concern in the literature [9]. This reflects an oversimplification of complex issues, which can fuel misinformation and conspiracy theories [88]. Oversimplified coverage may lead to superficial understanding and overconfidence in personal knowledge [88]. To counter this, future policies should clearly articulate the goals and rationale behind 20MNs in plain language, enabling the public to better understand and evaluate these interventions.

##### Liveability

Far-right sources also displayed status-quo bias regarding liveability, suggesting that change is unnecessary. However, pedestrian fatalities remain a serious concern. In the US, such deaths rose by 50% between 2013 and 2022, and by 5% in Great Britain between 2022 and 2023 [89, 90]. These figures highlight the need for enhanced safety measures and better infrastructure to improve urban liveability.

##### Goal framing

Goal framing and sociodemographic factors such as sex, age, and place of residence can shape public perceptions and acceptability of transportation policy [91]. This research suggests that current 20MN policy framing may reduce acceptability, though which framing causes this remains unclear. Future proposals should be intentionally designed and framed to improve public acceptance and support desired behaviour changes [91].

#### COVID-19 as an accelerator

Media coverage of 20MN criticisms/conspiracy theories increased significantly post-pandemic, many sharing similarities with COVID-19 conspiracy theories. These findings add to evidence linking 20MN conspiracy theories to the pandemic [12, 14]. Research also shows that non-COVID conspiracy theories such as anti-vaccine, QAnon, and 5G are also tied to the pandemic [92]. This analysis revealed links between 20MNs and unrelated conspiracies, suggesting shared themes and overlapping belief systems, which are explored further below.

#### Criticism or conspiracy theory?

The CDA showed that political groups have different thresholds for labelling ideas as conspiracy theories. Far-right media, while rejecting the “conspiracy theorist” label, contributed to disinformation, aligned with unrelated conspiracies, and promoted climate lockdown narratives without sufficient evidence. This does not suggest the far-left lacks conspiratorial beliefs, but 20MNs appear to be of particular concern to the far-right. Imhoff et al. [34] found that both far-left and far-right individuals are more likely to engage in black-or-white thinking, attributing societal issues to malicious actors. However, socially rather than economically conservative far-right individuals were especially prone to conspiracy thinking [34]. These findings suggest that similar cognitive processes inform conspiracy thinking, irrespective of ideological beliefs.

Douglas et al. [20] argue that people may turn to conspiracy theories to meet their epistemic needs when confronted with overwhelming, incomplete, or contradictory information about social and political events. Such beliefs may stem from a psychological need to regain control or give meaning to lived experiences [20]. People with high anxiety or low thresholds for threat perception are also more prone to conspiracy thinking [34, 93]. Once established, conspiracy beliefs are resistant to change, as contradictory evidence is viewed as untrustworthy [94].

Regardless of political affiliation or personal beliefs, there are also distinct personal, social and political motives for spreading conspiracy theories [20, 95]. Individuals may share conspiracies to achieve a sense of community, feel empowered, or denounce perceived injustice and corruption [95]. Public figures may have financial incentives to monetize conspiracies through crowdfunding, media sales, merchandising, or promoting alternative health products [95]. Political groups and entities may perpetuate conspiracy theories to garner support, justify actions or inactions, or divert public attention from other societal issues [96].

In the context of 20MNs, these mechanisms intersect with material and power concerns. Conspiracy theories often frame 20MNs, LTNs, and other urban planning interventions as top-down policies by tyrannical governments that threaten freedoms, economic stability, agency, and autonomy. The prevalence of such narratives may indicate that these policies have not been adequately communicated or explained to the public. These beliefs can shape public attitudes and contribute to resistance towards well-intentioned planning interventions.

##### Climate lockdowns

Concerns about 20MNs often shift into conspiracy theory when restrictions are perceived as overly severe. For example, Oxford’s LTN policy aims to reduce car traffic in certain areas during specific times using CCTV and fines [97]. Though not formally linked to 20MNs, the concepts were conflated by residents and media. Although 15MC phrasing has since been removed due to backlash, existing Oxford policy documents do not suggest that people will be confined to districts or restricted from leaving their communities [69, 97]. CCTV use is transparently outlined and comparable to standard traffic enforcement [97]. Furthermore, no existing 20MN policy documents promote confinement or surveillance to control movement [98–101]. These findings suggest that climate lockdown narratives are likely driven by the belief that innocuous policies will inevitably lead to severe, harmful consequences [102].

##### Anti-vaccine

20MN criticisms/conspiracies were commonly associated with “anti-vaxxers” in far-left and mainstream media. Goldberg and Richey [103] found that anti-vaccine sentiment correlates with a general tendency toward conspiratorial beliefs. These findings, along with this analysis, suggest a plausible link between 20MN and anti-vaccine conspiracies, though further research is needed.

##### Climate change

20MN conspiracies were closely tied to climate change scepticism or denial, particularly in far-right media. This aligns with Hornsey’s [104] findings that political orientation is a stronger predictor of climate change belief than other sociodemographic factors, with conservatives expressing more scepticism than liberals. López [105] argues that the fossil-fuel industry actively promotes climate disinformation to shape public opinion and protect profits, including by funding right-wing media that stokes fear about climate action. The WHO also recognizes industry-driven disinformation as a commercial determinant of health [106]. Notably, both far-right sources reviewed in this study reported critically on 20MNs and appeared to have potential fossil fuel funding conflicts (see Appendix 18). This suggests the fossil fuel industry may also play a role in spreading 20MN criticisms and conspiracy theories.

##### Power dynamics

In addition to influencing public opinion and fuelling climate change scepticism/denial, 20MN conspiracy theories may function to protect the material interests and power structures of fossil fuel, automotive, suburban real estate development, and other powerful industries [105]. By framing 20MNs as threats to personal freedoms, these narratives delegitimize policy interventions, reduce public support, and ultimately help sustain car-centric and fossil-fuel dependent economies [105]. Conversely, discourse advocating for 20MNs can be understood as seeking to redistribute this power towards communities, positioning health, equity, and wellbeing as key priorities of urban planning. These competing narratives illustrate that 20MN discourse does not simply reflect ideological differences but serves to create, uphold, or challenge existing power dynamics.

### Policy & practice implications

This research highlights media and politics’ influence on public perceptions and policy acceptability [107]. 20MN criticisms/conspiracy theories reveal potential unintended consequences of urban planning and public health interventions [108]. It is essential to assess whether such policies may cause physical, mental, tangible, or perceived harm [108]. Dismissive labels like “quack” and “conspiracy theorist”, especially from those in power, may reinforce feelings of discrimination and entrench existing beliefs [109].

Policymakers, planners, researchers, and politicians must be mindful of the power of language [91]. With ongoing pandemic spillover effects on public health and urban planning, stakeholder engagement and community buy-in are critical [110–112]. While media can be informative and support public understanding, it can also spread misinformation, disinformation, and conspiracy theories [16]. Media literacy is a key protective factor against such narratives and serves as a foundation for broader health literacy [17].

### Future research

These findings reinforce the need for further research on how 20MNs impact health/social inequities, to reduce public uncertainty and limit the spread of un-evidenced conspiracy theories. This study may serve as a foundation for evaluating public and media perceptions of 20MNs and similar urban planning interventions. Future qualitative research could expand sample size, include more diverse sources, or use larger research teams to improve generalisability and reduce researcher subjectivity.

These results also highlight the importance of framing in urban planning [91]. Further research should explore how current 20MN messaging influences public acceptability. Quantitative studies could assess how different framing strategies, such as emphasising pedestrian safety over individual car use, affect perceptions based on political or sociodemographic factors [91]. These insights could inform more effective communication strategies for future interventions.

## Conclusion

Significant public funds are being invested in the planning and implementation of 20MNs worldwide, based on claims of health and equity benefits. However, empirical evidence supporting these claims remains limited. This uncertainty has allowed conspiracy theories, particulary among far-right media, to thrive, often seeming to build on widespread COVID-19 public health responses. Future 20MN discourse should attempt to engage meaningfully with arguments across the political spectrum, and research should focus on clarifying the health and equity impacts of 20MNs and examing how framing influences their public acceptability.

## Supplementary Information

Supplementary Material 1.

## Data Availability

All data generated or analysed during this study are included in this published article and its supplementary information files.

## Abbreviations

15MC: 15-Minute City
20MN: 20-Minute Neighbourhood
C40: C40 Cities Climate Leadership Group
CCTV: Closed-Circuit Television
CDA: Critical Discourse Analysis
LTN: Low-Traffic Neighbourhood
MBFC: Media-Bias Fact-Check
NYT: The New York Times
PRISMA: Preferred Reporting Items for Systematic Reviews and Meta-Analyses
RQ: Research Question
SDOH: Social Determinants of Health
SES: Socioeconomic Status
TA: Thematic Analysis
TOL: The Times of London/The Sunday Times
UK: United Kingdom
UN: United Nations
US: United States
WC: Walkable City
WEF: World Economic Forum
WHO: World Health Organization
