# Supplementary material for: 20-minute neighbourhoods, criticisms, conspiracy theories, and health: a critical discourse analysis
Source: BMC Public Health. 2025 Nov 21;25:4093. doi: 10.1186/s12889-025-25416-y (PMC12639678; doi:10.1186/s12889-025-25416-y)
Supplement: Supplementary file 1 — Supplementary Material 1. [file 12889_2025_25416_MOESM1_ESM.docx]

# Supplementary Material

## Appendix 1: Timeline of Relevant Events

This table illustrates a timeline of events potentially impacting 20MN discourse

| November 30 – December 12 2015 | COP 21 Paris, France (1) |
| --- | --- |
| December 2019 | Respiratory outbreak of unknown aetiology identified in Wuhan, China (2) |
| January 7 2020 | Novel coronavirus identified in Wuhan, China (3) |
| ~ February 2020 | Paris mayor, Anne Hidalgo announces 15MC plans in re-election campaign (4) |
| March 11 2020 | WHO declares COVID-19 pandemic (5) |
| June 3 2020 | World Economic Forum (WEF) launces “The Great Reset” (6) |
| June 28 2020 | Hidalgo re-elected as mayor of Paris (7) |
| June 2021 | Carlos Moreno appointed co-chair of the French Ministry of Culture’s scientific and technical network on architectures in extreme environments (8) |
| October 3 2022 | Carlos Moreno/15MC Project wins “The United Nations-Habitat Scroll of Honour Award” (8) |
| December 31 2022 | Jordan Peterson tweets opposing 20MN and WEF (9) |
| February 9 2023 | UK - Conservative MP Nick Fletcher refers to 20MN as an “international socialist concept” that would “take away personal freedom” in the Commons (10) |
| February 18 2023 | Oxford, UK - Low Traffic Neighbourhood (LTN) and 20MN protest (11) |
| May 5, 2023 | WHO declares end to COVID-19 as a global health emergency (12) |
| October 2 2023 | UK - Conservative MP/transport secretary Mark Harper says “I am calling time on the misuse of so-called 15-minute cities”(13) |
| Early March 2024 | Oxford drops term 15MC from planning (14) |
| May 28-30 2024 | Canada - Protestors speak out against 15MC plans at Edmonton public hearings (11) |

## Appendix 2: Search Terms

This table illustrates the search terms utilized.

| **Search Terms** | |
| --- | --- |
| - “15-minute city” - “15-minute cities” - “20-minute neighbourhood” - “20-minute neighbourhoods” - “20-minute neighborhood” - “20-minute neighborhoods” - “30-minute territory” - “30-minute territories” | - “Walkable city” - “Walkable cities” - “X-minute city” - “X-minute cities” - “X-minute neighbourhood” - “X-minute neighbourhoods” - “X-minute neighborhood” - “X-minute neighborhoods” |

## Appendix 3: UK Publications

This table outlines the political leanings, factual reporting and credibility rankings, and traffic levels for each UK publication.

| **Publication** | **MBFC Political Leaning** | **MBFC Factual Reporting Rating** | **MBFC Credibility Rating** | **MBFC Traffic Levels** | **Additional Rationale for Selection** |
| --- | --- | --- | --- | --- | --- |
| **Times of London (TOL)** | Mainstream (right-centre biased - slight conservative political lean) (15) | High | High | High | Ranks highly in popularity, reputation, and breadth of topics covered (16) |
| **Novara Media** | Far-left biased (17) | Mixed | Medium | Medium | Reports being aligned with matters such as “the crisis of capitalism”, racism, and climate change (18) Perspectives of 20MNs focused on climate-change activism are relevant to the RQs. |
| **Spiked Magazine** | Right-biased (19) | Mixed | Medium | Medium | Claims to be “irreverent where others conform, questioning where others wallow in received wisdom, and radical where others cling to the status quo” (20) |

## Appendix 4: US Publications

This table outlines the political leanings, factual reporting and credibility rankings, and traffic levels for each UK publication.

| **Publication** | **MBFC Political Leaning** | **MBFC Factual Reporting Rating** | **MBFC Credibility Rating** | **MBFC Traffic Levels** | **Additional Rationale for Selection** |
| --- | --- | --- | --- | --- | --- |
| **New York Times (NYT)** | Mainstream left-centre biased - slight liberal political lean (21) | High | High | High | Ranks highly in popularity, reputation, and breadth of topics covered (16) |
| **Counterpunch** | Left-biased (22) | Mostly factual | High | Medium | Self-described “left-leaning perspective” to news reporting (23). Extra content contained exclusively in Counterpunch+, the premium subscription for this publication is not included |
| **The Federalist** | Far-right biased with questionable reporting based on “conspiracy theories, propaganda, and failed fact checks” (24) | Mixed | Low | High | Aligned with individual freedoms and traditionalist values (24). Has previously been criticized for spreading disinformation about COVID-19, this relates to RQ3 (25). |

## Appendix 5: Ownership and Funding

This table illustrates background, ownership, and funding information for each publication.

| **Publication** | **Est.** | **Ownership** | **Disclosed Funding Sources** | **Potential Funding Conflicts of Interest** | **Ads** | **Paywall** | **Subscription**  **Offered** | **Requests Donations** |
| --- | --- | --- | --- | --- | --- | --- | --- | --- |
| **Times of London** | 1785 | News Corp UK & Ireland (15) | Not publicly disclosed | None found | Yes | Yes | Yes | No |
| **Novara Media** | 2011 | Thousand Hands Ltd. (non-profit) (17,18) | Donations, YouTube revenue, grants, merchandise, and ticketed events (26) | Rosa Luxemburg Foundation - policy lobbying group supportive of democratic socialism (17,27) | No | No | No | Yes |
| **Spiked Magazine** | 2001 | Spiked Limited (19) | Donations (Spiked, n.d.), 70% reader funded (28) | Charles Koch Foundation, ties to the fossil-fuel industry/known for funding climate change disinformation (29,30) | Yes | No | No | Yes |
| **New York Times** | 1851 | Ochs-Sulzberger family via private shares (21) | Subscriptions and Advertising (31) | Previously accused of pushing the Ochs-Sulzberger family’s personal agendas through their reporting on certain topics (32) | Yes | Yes | Yes | No |
| **Counterpunch** | 1996 | Institute for the Advancement of Journalistic Clarity (non-profit) (22) | Donations and subscriptions for premium content (23) | None found | Minimal | No | Yes (premium content) | Yes |
| **The Federalist** | 2013 | FDRLST Media (24) | Not publicly disclosed | Funding from Ed Uihlein Family Foundation and DonorsTrust; both historically large donors to conservative causes and institutions (33) | Yes | No | Yes (ad free + commenting ability) | Yes |

***Appendix 6: External Relations***

This table provides detailed examples of external relations examined within each text.

| **External Relation** | **Description** | **Example** |
| --- | --- | --- |
| Social Relations | Relationships between groups and people | Influenced by political beliefs, socioeconomic status (SES), and social norms |
| Reciprocal Relations | Ways in which the texts might influence social practices and structures | Interacting with 20MN policy proposals positively or negatively. |
| Social Practices | Actions, routines, or ways of behaving | Automobile use/dependence, active transport use, use of local services. |

***Appendix 7: Internal Relations***

This table provides detailed examples of internal relations examined within each text.

| **Internal Relation** | **Description** | **Example** |
| --- | --- | --- |
| Apparent aims | What the text sets out to accomplish | Increase or decrease public support of 20MNs |
| Representations | Representations of social context, events, and actors | Stating that certain groups or people are fanatics, extremists, etc. |
| Positionality/Subject Positions | The specific language used that places people, events, or things within the context of a discourse or narrative (34). | The media may position a group of protestors as “an angry mob” or as “concerned citizens”, each phrase positions the group differently within the narrative. |
| Discursive framing | Frames are defined by Rushton and Williams (35) as: “linguistic, cognitive and symbolic devices used to identify, label, describe and interpret problems and to suggest particular ways of responding to them” (pg. 154). | Phrasing such as “pedestrian-friendly cities” vs. “anti-car cities”, each statement implies different problems and solutions in urban design. |

## Appendix 8: Pre-Pandemic Search Results

This table illustrates pre-pandemic search results.

| **Period** | **Pre-Pandemic**  **(March 10^th^, 2016 – March 10^th^, 2020)** | | | | | | | | | | | |  | | | |
| --- | --- | --- | --- | --- | --- | --- | --- | --- | --- | --- | --- | --- | --- | --- | --- | --- |
| **Search Terms** | **“15-minute city”/**  **“15-minute cities”** | | **“20-minute neighbourhood(s)"/**  **“20-minute neighborhood(s)”** | | **“Walkable city”/**  **“Walkable cities”** | | **“X-minute city”/**  **“X-minute cities”** | | **“X-minute neighbourhood(s)”/ “X-minute neighborhood(s)”** | | **“30-minute territory”/ “30-minute territories”** | |  |  |  |  |
| **Results** | **Total** | **Included** | **Total** | **Included** | **Total** | **Included** | **Total** | **Included** | **Total** | **Included** | **Total** | **Included** | **Total Results** | **Total Included** | **% Meeting inclusion criteria** |  |
| **New York Times** | **0** | **0** | **0** | **0** | **13** | **0** | **0** | **0** | **0** | **0** | **0** | **0** | **13** | **0** | **0** |  |
| **The Times of London** | **1** | **0** | **1** | **0** | **14** | **0** | **0** | **0** | **0** | **0** | **0** | **0** | **16** | **0** | **0** |  |
| **Counterpunch** | **0** | **0** | **0** | **0** | **0** | **0** | **0** | **0** | **0** | **0** | **0** | **0** | **0** | **0** | **0** |  |
| **Novara Media** | **0** | **0** | **0** | **0** | **0** | **0** | **0** | **0** | **0** | **0** | **0** | **0** | **0** | **0** | **0** |  |
| **The Federalist** | **0** | **0** | **0** | **0** | **0** | **0** | **0** | **0** | **0** | **0** | **0** | **0** | **0** | **0** | **0** |  |
| **Spiked Magazine** | **0** | **0** | **0** | **0** | **0** | **0** | **0** | **0** | **0** | **0** | **0** | **0** | **0** | **0** | **0** |  |
| **Total** | **1** | **0** | **1** | **0** | **27** | **0** | **0** | **0** | **0** | **0** | **0** | **0** | **29** | **0** | **0** |  |

## Appendix 9: Post-Pandemic Search Results

This table illustrates post-pandemic search results.

| **Period** | **Post-Pandemic**  **(March 11^th^, 2020 – March 11^th^, 2024)** | | | | | | | | | | | |  | | |
| --- | --- | --- | --- | --- | --- | --- | --- | --- | --- | --- | --- | --- | --- | --- | --- |
| **Search Terms** | **“15-minute city”/**  **“15-minute cities”** | | **“20-minute neighbourhood(s)"/**  **“20-minute neighborhood(s)”** | | **“Walkable city”/ “Walkable cities”** | | **“X-minute city”/**  **“X-minute cities”** | | **“X-minute neighbourhood(s)”/ “X-minute neighborhood(s)”** | | **“30-minute territory”/ “30-minute territories”** | |  |  |  |
| **Results** | **Total** | **Included** | **Total** | **Included** | **Total** | **Included** | **Total** | **Included** | **Total** | **Included** | **Total** | **Included** | **Total Results** | **Total Included** | **% Meeting inclusion criteria** |
| **New York Times** | **16 *(2)** | **5** | **0** | **0** | **24 *(3)** | **0** | **0** | **0** | **0** | **0** | **0** | **0** | **40 *(5)** | **5** | **14.29%** |
| **The Times of London** | **52 *(7)** | **8** | **28 *(6)** | **2** | **22** | **1** | **0** | **0** | **0** | **0** | **0** | **0** | **102*(13)** | **11** | **12.36%** |
| **Counterpunch** | **6* (1)** | **1** | **0** | **0** | **3** | **0** | **0** | **0** | **0** | **0** | **0** | **0** | **9 *(1)** | **1** | **12.5%** |
| **Novara Media** | **4* (1)** | **2** | **2 *(2)** | **0** | **0** | **0** | **0** | **0** | **0** | **0** | **0** | **0** | **6 *(3)** | **2** | **66.66%** |
| **The Federalist** | **6 *(2)** | **2** | **0** | **0** | **1** | **0** | **0** | **0** | **0** | **0** | **0** | **0** | **7 *(2)** | **2** | **40%** |
| **Spiked Magazine** | **15 *(3)** | **7** | **0** | **0** | **0** | **0** | **0** | **0** | **0** | **0** | **0** | **0** | **15*(3)** | **7** | **58.33%** |
| **Total** | **99** | **25** | **30** | **2** | **50** | **1** | **0** | **0** | **0** | **0** | **0** | **0** | **179 *(27)** | **28** | **18.42%** |

* Duplicate result(s) removed

## Appendix 10: Code Examples

This table illustrates code examples identified in the critical discourse analysis.

| **Stance** | **Code** | **Description** | **Example** |
| --- | --- | --- | --- |
| Supportive | Transportation | 20MNs promote walking, cycling, and public transit. | *“Proponents say 15-minute cities are healthier for their residents and for the environment, as they encourage walking and discourage a reliance on cars.”* |
| Against | Climate  Lockdowns | 20MNs will result in ‘climate lockdowns’ | *“I’m going to say it: this is a climate lockdown. It is perfectly legitimate to describe top-down, eco-justified restrictions on people’s freedom to drive as a climate lockdown.”* |
| Mixed  /Unsure | Impractical | 20MNs may not be practical | *“Walking and biking are good for us and pleasurable but are few people’s choice on a cold wet day. Aren’t 15-minute cities best located in the Mediterranean and South America?”* |

## Appendix 11: Full Code Book

This table is the full exported code book from Nvivo 15 with examples.

|  | **Code** | **Description** | **Example** |
| --- | --- | --- | --- |
|  | Anti-Car | An attack on cars | *“There really is a war on the car. From driving taxes to Low Traffic Neighbourhoods to the proposed ‘15-minute city’, the great liberty of the motorcar is being sacrificed to the carboncutting mania of elites who have lost faith in modernity.”* |
|  | Anti-Vaccine **(Deductive)** | Criticisms/conspiracy theories are linked to anti-vaccine beliefs | *“Anti-vaccination conspiracy theorists latched on to the [15MC] phrase at the start of last year, wrongly framing it as a plan to control people’s movements.”* |
|  | Anti-Working Class | Disproportionately harm working class people | *“The elites are hell-bent on restricting car-use, and this will make life harder for people, especially working-class people.”* |
|  | Cars as Freedom | Cars give people freedom | *“‘The car is unique – it gives human beings a mandate to go wherever they want, whenever they want’, said Pehr Gyllenhammar, CEO of Volvo from 1970 to 1994. We once celebrated that mandate.”* |
|  | Climate Change Denial **(Deductive)** | Rejecting that climate change exists or is a problem | *“‘Question any aspect of the climate-alarmist agenda, including the harebrained claim that billions will soon die in a fiery apocalypse of man’s making, and you’ll be branded with that D-word. It marks you out as unfit for public life.”* |
|  | Climate Lockdowns | Will result in ‘climate lockdowns’ like COVID-19 lockdowns | *“I’m going to say it: this is a climate lockdown. It is perfectly legitimate to describe top-down, eco-justified restrictions on people’s freedom to drive as a climate lockdown.”* |
|  | Concentration Camps | Likened to Nazi concentration camps | *“there’s a popular meme on Twitter/X that runs, “They call them ‘15-minute cities’ because if they were called ‘concentration camps’, no one would want to live in them.’”* |
|  | Confinement | People will be forced to stay within their 20MN/district | *“Do not leave your allotted zone, at least most of the time – that is the policy. Or it could soon be after Oxfordshire County Council decides on the matter on 29 November.”* |
|  | Conspiracies are Harmful | Conspiracy theories are harmful to people and populations | *“‘These conspiracy theories are causing real-world harm and need to stop,’ Liz Leffman, the leader of the Oxfordshire County Council, said in a video. ‘We have been receiving many calls and emails from worried residents in genuine fear that they might be locked in their own homes. This is categorically untrue.’”* |
|  | Covid Accelerated Conspiracy Theories **(Deductive)** | Conspiracy theories are linked to COVID-19 | *“Conspiracies about the ‘great replacement theory’, ‘15-minute cities’ and central bank digital currencies are gaining traction as a fringe newspaper attempts to capitalise on the post-pandemic opportunity.”* |
|  | Covid Accelerated 20MNs **(Deductive)** | Changes in habits resulting from the pandemic accelerated 20MNs | *“What accelerated the idea’s implementation by cities round the world, however, was Covid. Many found they could work at home quite easily and, freed from hellish commutes, fell back in love with their local environments”* |
|  | Criticisms are anti-urban | Criticisms and conspiracies about 20MN are against/blame societal issues on urban populations/inhabitants | *“I’ve noted before that there’s an unwritten rule in American politics that it’s OK for politicians to disparage big cities and their residents in a way that would be considered unforgivable if anyone did the same for rural areas.”* |
|  | Digital Accessibility | Enhanced access to digital services should be utilized in addition to improving physical proximity | *“Developing more 20-minute neighbourhoods across Scotland may also need to be adapted for the digital future, Olsen said. ‘Covid accelerated the idea that physical access isn’t everything, and we need to think about what we access digitally as well.’”* |
|  | Dystopian Future | Will lead to a dystopian or totalitarian future | *“Days later, Mark Dolan, a host on GB News, a Fox News-style TV channel that began last year, warned viewers of what he called the ‘dystopian plan’ being pursued by several communities that depends on ‘a surveillance culture that would make Pyongyang envious.’”* |
|  | Economic Punishment | 20MNs/traffic reduction measures financially punish those who don't or can't comply with fines | *“That is, it’s a kind of sin tax, to use John Stuart Mill’s phrase, where you’ll be fined for the sin of driving in the hope that you’ll eventually feel so economically punished that you’ll choose walking instead.”* |
|  | Gaslighting | Legitimate concerns about 20MNs are unfairly being invalidated as conspiracy theories | *“Yet the hysterical denunciation of pro-car protesters as maniacs and conspiracy theorists who are one car journey away from becoming open fanboys of the Fourth Reich is a new low. It’s classic gaslighting”* |
|  | Ghettos | Will ghettoize/segregate communities | *“Those are likely to be used to promote densified housing along the lines of the “15-minute city” (more accurately termed 15-minute ghettoes)-”* |
|  | Greenwashing | Used to make governments/cities appear more environmentally conscious than they are | *“If a developer is so intent on cutting unsustainable travel, it is hypocritical to pop up in an out-of-town location. It smacks of greenwashing -”* |
|  | Impractical | May not be practical to implement in many places | *“Walking and biking are good for us and pleasurable but are few people’s choice on a cold wet day. Aren’t 15-minute cities best located in the Mediterranean and South America?”* |
|  | Improve Health  **(Deductive)** | Will improve the physical health of populations | *“The Scottish government has made a commitment to apply the concept nationally, focusing particularly on deprived areas to ‘encourage healthier lifestyles and reduce carbon emissions’”* |
|  | Irony | Conspiracy theories are ironic | *“And as usual the people who yell loudest about ‘freedom’ are actually the ones who want to practice coercion, preventing other Americans from living in ways they disapprove of.”* |
|  | Liberties | Impede personal liberties and freedoms | *“A member of Britain’s Parliament said that 15-minute cities were “an international socialist concept” that would ‘cost us our personal freedoms.’”* |
|  | Liveability | Make communities more suitable to live in | *“In responding to the challenges posed by the pandemic and climate change, a number of cities around the world have adopted policies inspired by the 15-minute model, aiming to improve their livability and sustainability.”* |
|  | Pandemic Recovery | Help/helped communities recover from the negative effects of the pandemic | *“A group of nearly 100 mayors worldwide embraced [15MCs] as a way to help recover from the pandemic.”* |
|  | Political Affiliation | Stance on 20MNs is linked to political beliefs | *“15-minute cities are, depending on your political affiliations, either a) a perfectly sensible piece of urban planning that makes places more pedestrian-friendly, or b) THE BEGINNING OF BIG BROTHER’S OPEN-AIR PRISONS/DEATH CAMPS.”* |
|  | Quackery | Conspiracy theories are dishonest and outlandish | *“It’s such a laughably mild and practical suggestion that seeing it co-opted into the Portfolio of Online Conspiracy Theories — alongside antivax and 5G mast protests — is like finding out that Donald Trump’s latest bugbear is the Blue Peter dog. Which could, of course, happen.”* |
|  | Quality of Life | Will make life better/improve mental health | *“Proponents of the 15-minute city think it will make us happier, too, as we get to know our neighbors instead of rushing from one thing to the next.”* |
|  | Quality of Services | Proximity to services alone is not sufficient to improve health, quality and type of services needs to be considered | *“Quite often in these deprived areas there is the co-location of health benefiting and damaging facilities, such as a high density of fast-food, gambling, alcohol and tobacco outlets. Bruntsfield has fewer of these health damaging facilities in a well-connected, affluent 20-minute neighbourhood. So I think policy going forward needs not just to think about access, but quality access.”* |
|  | Reduce Health Inequities **(Deductive)** | Decrease existing heath/social inequities | *“- a report from C40 Cities, a group of 96 cities around the world working to mitigate the effects of climate change, that said “any city where a private vehicle is necessary to get around is likely to be fundamentally unequal.”* |
|  | Resiliency  /Sustainability | Make communities more resilient to the negative impacts of climate change | Under *“This is How Britain Can Actually Prepare for Extreme Weather”* - “*Rethink transportation and embrace 15-minute cities.”* |
|  | Restriction of Choice | Restrict people's ability to choose where, how, and when they shop, travel, socialize, etc. | *“Sure, the ‘traffic filters’ and the ‘15-minute city’ are, strictly speaking, separate policies, but both have been embraced by Oxford, both are designed to limit car use, and both will impact on people’s freedom of choice and freedom of movement.”* |
|  | Restriction of Movement | Restrict the free movement of populations | *“On the surface, these 15-minute neighbourhoods might sound pleasant and convenient. But there is a coercive edge. The council plans to cut car use and traffic congestion by placing strict rules on car journeys.”* |
|  | Rural Residents | Rural populations will be forced to live in 20MNs | *“QAnon supporters said the derailment of a train carrying hazardous chemicals in Ohio was an intentional move meant to push rural residents into 15-minute cities.”* |
|  | Social Engineering | An attempt by government to manipulate and influence social behaviours and structures | *“And the aim really is to socially re-engineer the city’s populace out of using their cars –”* |
|  | Surveillance | Increased surveillance/ reduce privacy of populations | *“These 15-minute smart cities will maintain watch and control over the populace’s movement via sophisticated surveillance technology that is already being installed across the world -”* |
|  | The Elite | Will only benefit the elite/wealthy in society | *“Nothing better speaks to class privilege these days than hatred for the car and love for the ‘15-minute city’. It is a kind of bourgeois mimicry of the plain lives people led in the era before cheap food and mass car ownership.”* |
|  | Transportation | Promote walking, cycling, and public transit | *“Proponents say 15-minute cities are healthier for their residents and for the environment, as they encourage walking and discourage a reliance on cars.”* |
|  | WEF | Conspiracy theories are linked to the World Economic Forum's "great reset" proposal | *“The concept of 15-minute cities has also been caught up in broader conspiracy theories about efforts to remake society as the world emerges from the pandemic. The focus of many of those theories is an effort by the World Economic Forum called ‘The Great Reset.’”* |
|  | Wokeness | 20MNs belong to “woke culture” | *“Unfortunately, our government’s relentless push for woke policies tells us that we cannot expect to understand those terms as traditional virtues.”* |
|  | Worsen Health Inequities **(Deductive)** | Worsen existing or create new health/social inequities | *“- we shouldn’t forget the needs of older citizens, those with disabilities and children – as well as the women who so often look after them.”* |
|  | Zoning | Single-use zoning impedes 15MC/20MN/WCs | *“Making walkable cities possible requires both loosening and tightening restrictions on urban development: Localities would have to allow more construction of multifamily housing and multistory buildings, while restricting car traffic in certain areas.”* |

# Additional Supplementary Material

## **Appendix 12: The New York Times Results**

This table illustrates all *New York Times* search results

| **The New York Times** | |
| --- | --- |
| Search terms: “15-minute city”/ “15-minute cities” | |
| **Pre-Pandemic** | **Post Pandemic** |
| **Included** | **Included** |
| N/A | 1. The Pandemic Emptied Europe’s Cities. What Will Bring People Back? - February 11, 2021 2. How One San-Francisco Street Survived the Pandemic - October 11, 2021 3. The 15-Minute City: Where Urban Planning Concepts Meets Conspiracy Theories - March 1, 2023 4. City Life, Culture Wars and Conspiracy Theories - March 6, 2023 5. He Wanted to Unclog Cities, Now He’s ‘Public Enemy No. 1’ - March 28, 2023 |
| **Excluded** | **Excluded** |
| N/A | 1. A South African Shopping Site Where Artisans and Community Meet - Nov. 24, 2021 **(irrelevant)** 2. A Key to Controlling Emissions: More Buildings in a City's Unused Spaces – September 19, 2022 **(irrelevant)** 3. 6 Cities on 5 Continents That Are Reimagining Urban Life – March 16, 2023 **(irrelevant)** 4. Obamacare Keeps Winning - March 29, 2023 **(newsletter/irrelevant)** 5. A towering, Terrifying Demon Horse Isn’t Even the Weirdest Part - April 29, 2023 **(irrelevant)** 6. Parisians Are Pledging Allegiance to the ‘Republic of Super Neighbors.’ They Must Bring Cheese - August 30, 2023 **(irrelevant)** 7. 36 Hours in Glasgow - Oct 19, 2023 **(travel guide)** 8. How an L.A. Rams Training Complex Could Help Transform Urban Sprawl - Nov 23, 2023 **(irrelevant)** 9. The ZIP Code Shift: Why Many Americans No Longer Live Where They Work – March 4, 2024 **(irrelevant)** |
| Search Term: “20-minute neighbo(u)rhood”/ “20-minute neighbo(u)rhood” | |
| **Pre-Pandemic** | **Post-Pandemic** |
| **Included** | **Included** |
| N/A | N/A |
| **Excluded** | **Excluded** |
| N/A | N/A |
| Search Term: “Walkable city”/ “Walkable cities” | |
| **Pre-Pandemic** | **Post-Pandemic** |
| **Included** | **Included** |
| N/A | N/A |
| **Excluded** | **Excluded** |
| 1. The Future of Retirement Communities: Walkable and Urban – October 14, 2016 **(irrelevant)** 2. How Sicily Cemented an Unlikely Friendship – April 18, 2017 **(travel)** 3. How Safer Streets Can Thwart Terrorists – November 3, 2017 **(irrelevant)** 4. A Smorgasbord of Solutions for Global Warming – April 25, 2018 **(irrelevant)** 5. 36 Hours in Victoria, British Columbia – April 12, 2018 **(travel guide)** 6. The Strange, Enduring Appeal of Biarritz – May 14, 2018 **(travel)** 7. Francine Prose on Montreal in the Spring: The Time of Butterflies – June 11, 2018 **(travel)** 8. 36 Hours in Chamonix – December 13, 2018 **(travel)** 9. DealBook Briefing: Big Tech’s Tough Day in D.C. - July 17, 2019 **(irrelevant)** 10. 36 Hours in Hobart (and Environs) - December 5, 2019 **(travel guide)** 11. Private Parking Goes Deluxe – September 13, 2019 **(real estate)** 12. 36 Hours in Dublin – September 26, 2019 **(travel guide)** 13. Why Midsize Cities Struggle to Catch Up to Superstar Cities – July 16, 2019 **(irrelevant)** | 1. A Renter Tests His Budget in Connecticut. Which of These Homes Would You Choose? - May 14, 2020 **(real estate)** 2. As Some New Yorkers Flee, Others Move Closer to the Office – September 4, 2020 **(irrelevant)** 3. Which Cities Are Best for Walking Your Dog? - March 3, 2022 **(irrelevant)** 4. Why It’s So Hard to Get Cars Off the Road – June 9, 2022 **(irrelevant)** 5. ‘The Biggest Uncertainty Is Us’ – June 24, 2022 **(newsletter)** 6. Alabama Takes from The Poor and Gives to the Rich – July 27, 2022 **(irrelevant)** 7. 36 hours in Madrid - February 16, 2023 **(travel guide)** 8. Awash in Asphalt, Cities Rethink Their Parking Needs - March 7, 2023 **(irrelevant)** 9. When a Walkable City Becomes a Death Trap – April 28, 2023 **(irrelevant)** 10. 36 hours in Bath, England – June 15, 2023 **(travel guide)** 11. The Art of Being a Flâneur – June 19, 2023 **(travel)** 12. 36 hours in Palermo, Italy – August 17, 2023 **(travel guide)** 13. How the Dream of Building a California City From Scratch Got Started – August 31, 2023 **(irrelevant)** 14. 36 hours in Córdoba, Spain – December 21, 2023 **(travel guide)** 15. Saratoga Springs, N.Y.: An Urban Oasis at the Foot of the Adirondacks - September 13, 2023 **(real estate)** 16. Living in ... Saratoga Springs, N.Y. - September 13, 2023 **(real estate)** 17. How the Monotony of Sunny California Made New York Shine – July 31, 2023 **(real estate)** 18. Stuffy, Preppy, Sleepy: Can a Rebrand Fix Connecticut’s Reputation? – December 9, 2023 **(irrelevant)** 19. How Can Buildings Beat the Heat in a Desert City? Blend Ancient and Modern – December 9, 2023 **(irrelevant)** 20. Why Are American Drives So Deadly? – January 10, 2024 **(irrelevant)** 21. The Surprising Left-Right Alliance That Wants More Apartments in Suburbs – March 9, 2024 **(irrelevant)** |

## Appendix 13: Counterpunch Results:

This table illustrates all *Counterpunch* search results

| **Counterpunch** | |
| --- | --- |
| Search terms: “15-minute city”/ “15-minute cities” | |
| **Pre-Pandemic** | **Post Pandemic** |
| **Included** | **Included** |
| N/A | - - - 1. Right Wing Panic Over ‘15-Minute Cities’ About Freedom for Cars, Not People – February 24, 2023 |
| **Excluded** | **Excluded** |
| N/A | - - - 1. After Elections, Seoul Braces for Environmental Inaction – April 16, 2021**(irrelevant)**       2. In a Manichean as Metapolitical Framework Real Climate Change has only One ‘Cui Bono?’ Alas. -September 15, 2023 **(irrelevant)**       3. Yves Engler, Author at CounterPunch.org **(author page)**       4. Individualism is Killing the Planet – August 8, 2023 **(irrelevant)** |
| Search Term: “20-minute neighborhood”/ “20-minute neighborhoods” | |
| **Pre-Pandemic** | **Post-Pandemic** |
| **Included** | **Included** |
| N/A | N/A |
| **Excluded** | **Excluded** |
| N/A | N/A |
| Search Term: “Walkable city”/ “Walkable cities” | |
| **Pre-Pandemic** | **Post-Pandemic** |
| **Included** | **Included** |
| N/A | N/A |
| **Excluded** | **Excluded** |
| N/A | 1. Why Must the People Protest? – June 18, 2020 (irrelevant) 2. Roaming Charges: The Pitch of Frenzy – September 8, 2023 (irrelevant) 3. The New Fight for Mobility Justice – September 16, 2022 (irrelevant) |

## Appendix 14: The Federalist Results

This table illustrates all *The Federalist* search results

| **The Federalist** | |
| --- | --- |
| Search terms: “15-minute city”/ “15-minute cities” | |
| **Pre-Pandemic** | **Post Pandemic** |
| **Included** | **Included** |
| N/A | 1. To Address The Loneliness Epidemic, The Feds Want To Control Your Town And Friends – May 30, 2023 2. Anti-Air Travel Climate Psychos Want To Abolish Freedom Of Movement – October 6, 2023 |
| **Excluded** | **Excluded** |
| N/A | 1. TikTok Deleted My Video Exposing Climate Cultists’ Dystopian Plan To Restrict Freedom Of Movement - October 20, 2023 **(irrelevant)** 2. The Federal ‘Kill Switch’ Signals Our Surrender To Tech Overlords – November 13, 2023 **(irrelevant)** |
| Search Term: “20-minute neighborhood”/ “20-minute neighborhoods” | |
| **Pre-Pandemic** | **Post-Pandemic** |
| **Included** | **Included** |
| N/A | N/A |
| **Excluded** | **Excluded** |
| N/A | N/A |
| Search Term: “Walkable city”/ “Walkable cities” | |
| **Pre-Pandemic** | **Post-Pandemic** |
| **Included** | **Included** |
| N/A | N/A |
| **Excluded** | **Excluded** |
| N/A | - - - 1. GOP’s Unifying Midterm Strategy Should Treat Democrats’ Green Agenda As Culture War – September 9, 2022 **(irrelevant)** |

## Appendix 15: The Times of London Results

This table illustrates all *The Times of London* search results

| **The Times of London** | |
| --- | --- |
| Search terms: “15-minute city”/ “15-minute cities” | |
| **Pre-Pandemic** | **Post Pandemic** |
| **Included** | **Included** |
| N/A | 1. Waltham Forest, the suburb that pioneered the ‘20‑minute neighbourhood’ - September 20, 2020 2. What are 15-minute cities and why are antivaxxers so angry about them? - February 22, 2023 3. Is the 15-minute city the future of urban living? - February 26, 2023 4. Scottish minister accused of pushing 15-minute cities conspiracy theory – October 3, 2023 5. The new Tory conspiracy theory? A 15-minute stroll is ‘sinister’ - October 28, 2023 6. Meet Carlos Moreno, the man who ignited the car culture wars – February 29, 2024 7. Oxford planners drop “toxic” 15-minute city phrase – March 6, 2024 8. Antivax newspaper claims to print 150,000 copies a month – June 16th, 2023 |
| **Excluded** | **Excluded** |
| 1. Kevin Pringle: Early lessons of Covid-19 — travel less and use more tech – March 7^th^, 2020 **(irrelevant)** | 1. Reward people to gamify green behaviour, says executive – November 27, 2020 **(irrelevant)** 2. Where to buy property in Paris, France - December 4, 2020 **(real estate)** 3. Could turning shops into homes save our high streets? - February 26, 2021**(irrelevant)** 4. Cars to be driven from the streets in new Vienna village - March 12, 2021**(irrelevant)** 5. The word on the street is Al Fresco - April 11, 2021**(food)** 6. Sir George Iacobescu: man who put Canary Wharf on the map steps down from executive role - June 4, 2021**(irrelevant)** 7. Artists come out to fight for 1916 cityscape - July 11, 2021**(irrelevant)** 8. On a roll: how Utrecht became the world’s best city for cycling - August 27, 2021**(irrelevant)** 9. Engineer casts doubt on Galway ring road - September 27, 2021 **(irrelevant)** 10. Where to buy property in Battersea, London - October 1, 2021 **(real estate)** 11. Developers are off to see if City Edge will be a wizard idea - October 30, 2021 **(irrelevant)** 12. IPUT poised to put another €250m into Carrickmines - January 9, 2022 **(irrelevant)** 13. “Dwindling infrastructure’ leaves Dublin in need of new arts centre - April 14, 2022 **(irrelevant)** 14. How Brent Cross is reinventing urban living for the modern age - April 28, 2022 **(irrelevant)** 15. Oxford set to cut its famous traffic jams by degrees - October 23, 2022 **(irrelevant)** 16. Westfield chief Jean-Marie Tritant: Online can’t beat real shops - October 29, 2022 **(irrelevant)** 17. Energy crisis could cripple high street, says Mary Portas – November 9, 2022 **(irrelevant)** 18. London is exhausting, so we moved to Vigo in Spain - January 10, 2023 **(irrelevant)** 19. How Athens is turning its disused airport into a smart city - January 29, 2023 **(irrelevant)** 20. Which city is better to live in: Oxford or Cambridge? - March 26, 2023 **(real estate)** 21. E-Scooters – Paris has banned them. Is it time that we did too? - April 5, 2023 **(irrelevant)** 22. Why more Brits are choosing Portugal for their retirement - April 26, 2023 **(real estate)** 23. Starmer’s populist plan: shut up about green stuff, talk cost of living - August 13, 2023 **(irrelevant)** 24. Give everyone a right to grow trees, says government tsar - September 26, 2023 **(irrelevant)** 25. Monica Ali: ‘I lie in the bath until it goes cold, thinking about storylines’ - November 9, 2023 **(interview)** 26. Sadiq Khan’s ever-expanding Ulez leaves Home Counties fuming – February 12th, 2023 **(irrelevant)** 27. Foolproof by Sander van der Linden review — how to combat fake news: an expert’s guide – March 3rd, 2023 **(irrelevant/link to different article)** 28. The real AI thread: how powerless it makes us – April 15th, 2023 **(irrelevant/brief mention of 15MCs)** 29. Anti-Ulez protestors warn of French-style unrest as London cameras vandalised – May 25th, 2023 **(irrelevant/brief mention of 15MCs)** 30. Pay to park anywhere in country on a single app – September 30th, 2023 **(irrelevant/brief mention of 15MCs)** 31. Parisians get to know their neighbours with Sunday lunch for 1,000 – September 30th, 2023 **(irrelevant)** 32. The Times view on Conservative Party conference: Sunak’s Challenge – October 2nd, 2023 **(irrelevant)** 33. Sunak’s appeal is lost to half the electorate – October 3rd, 2023 **(irrelevant)** 34. These days the ‘gay’ Tory lion would be on the first flight back to Africa – October 6th, 2023 **(irrelevant)** 35. Make do and mend offers a handy fix to get our cities working again – October 13th, 2023 **(irrelevant/brief mention of 15MCs)** 36. How to build a new town — lessons from Milton Keynes – October 13th, 2023 **(irrelevant)** 37. Alex Batty: How the Pyrenees became a haven for cults and hippies – December 23rd, 2023 **(irrelevant)** |
| Search terms: “20-minute neighbourhood” / “20-minute neighbourhoods” | |
| **Pre-Pandemic** | **Post Pandemic** |
| **Included** | **Included** |
| N/A | 1. Dunbar developers’ ‘Eco plans’ are just greenwashing – October 23, 202 2. The best 20-minute neighbourhoods in Scotland – December 4, 2022 |
| **Excluded** | **Excluded** |
| 1. Best places for a car-free commute in Britain: from Bristol to Liverpool, Newcastle and Nottingham – September 22, 2019 **(irrelevant)** | 1. Coronavirus in Scotland: Scots face bumpy ride ahead after cases rise to lockdown levels – September 2, 2020 **(irrelevant)** 2. Big Sister Sturgeon is shrinking our horizons – September 6, 2020 **(irrelevant)** 3. Cities are dead now but they’ll be back and some will be smarter – September 20, 2020 **(irrelevant)** 4. Transport hub plan to revitalise town centres – February 15, 2021 **(irrelevant)** 5. Away from our screens, it’s friends we need – March 9, 2021 **(irrelevant)** 6. Revealed: the deadly toll from Scotland’s polluted air – July 17, 2021 **(broken link)** 7. Motorists to be driven from city centres in Scotland under plan to cut emissions – October 23, 2021 **(irrelevant)** 8. Minister could put A-road dualling plan into reverse – November 11, 2021 **(irrelevant)** 9. West Town plan for new homes to ease housing crisis in Edinburgh – December 3, 2021 **(irrelevant)** 10. Green housing schemes have nowhere to park your car – November 13^th^, 2021 **(irrelevant)** 11. A city must never grow tired of regeneration – March 28, 2022 **(irrelevant)** 12. Cycle lanes ‘make bus use less attractive’ – May 2, 2022 **(irrelevant)** 13. Will Edinburgh’s uneasy alliance prove a capital idea again? – May 3, 2022 **(irrelevant)** 14. Ditching private vehicle for car sharing ‘saves thousands’- July 4, 2022 **(irrelevant)** 15. The ten best new-build neighbourhoods – October 7^th^, 2022 **(irrelevant)** 16. Glasgow streets to be made with women in mind – October 28, 2022 **(irrelevant)** 17. We have squandered our Victorian legacy – May 3, 2023 **(irrelevant)** 18. Former Orkney baker serves up call for islands to leave UK – July 7, 2023 **(irrelevant)** 19. Voters won’t accept green policies unless they make sense and are fair – July 23, 2023 **(irrelevant)** 20. Edinburgh fast-tracks plans to drive cars off the streets – January 29, 2024 **(irrelevant)** |
| Search terms: “Walkable city”/ “Walkable cities” | |
| **Pre-Pandemic** | **Post-Pandemic** |
| **Included** | **Included** |
| N/A | 1. Paris braces for a ‘big bang’ if Anne Hidalgo wins as mayor again – June 28, 2020 |
| **Excluded** | **Excluded** |
| 1. How to do Boston on a shoestring – September 9, 2016 **(travel)** 2. Where to go for a quick pre-Christmas escape – November 18, 2016 **(travel)** 3. Welcome to India’s secret golden triangle – January 17, 2017 **(travel)** 4. Edinburgh going extra mile to cut car numbers – April 3, 2018 **(irrelevant)** 5. Hit the road – January 25, 2019 **(travel)** 6. We want a family adventure in Costa Rica – August 26, 2017 **(travel)** 7. Seeking a quiet destination to spend Christmas – September 22, 2017 **(travel)** 8. The big weekend: Sofia – September 23, 2017 **(travel)** 9. Athens city guide: the Big Weekend – June 10, 2018 **(travel)** 10. A weekend in ... Zadar, Croatia – July 6, 2018 **(travel)** 11. Student city guide to Cambridge – September 22. 2018 **(irrelevant)** 12. Jerez: on the flamenco trail – January 19, 2019 **(travel)** 13. Dazzling delights of gorging in Georgia – May 11, 2019 **(travel)** 14. Election 2020: Ban idling cars outside school gates to cut air pollution, Green Party demand – January 17, 2020 **(irrelevant)** | 1. How Athens tried to end its love affair with the car – October 19, 2020 **(irrelevant)** 2. Why Covid made everyone want to move to Yorkshire – May 6, 2021 **(irrelevant)** 3. Douglas Stuart’s Glasgow is changing fast. Will Shuggie Bain feel at home? – May 17, 2021 **(irrelevant)** 4. How an entire London street got a mansard roof makeover – August 14, 2021 **(irrelevant)** 5. Where to buy property in Bordeaux, France – September 23, 2021 **(real estate)** 6. Planning guru Brent Toderian to aid birth of a new liveable city in Limerick – March 22, 2022 **(irrelevant)** 7. The beauty of the Baltic – April 21, 2022 **(travel)** 8. The big weekend: Manchester – April 21, 2022 **(travel)** 9. ‘Car hire mix-up left me £1,775 out of pocket’ – October 17, 2022 **(travel)** 10. Sofia city guide: your weekend break sorted – December 24, 2022 **(travel)** 11. Velvet Manchester hotel review: ‘The look is opulent and sophisticated’- December 31, 2022 **(travel)** 12. Why digital nomads are moving to Alicante, Spain – April 5, 2023 **(irrelevant)** 13. I moved abroad to survive the cost of living crisis. I’m not the only one – April 10, 2023 **(irrelevant)** 14. ‘My first-class airline seat wouldn’t recline — can I get a refund?’ – June 18, 2023 **(travel)** 15. Six Spanish city breaks that are overlooked in winter – December 2, 2023 **(travel)** 16. The billionaires’ dream city that’s a nightmare for Californians – December 9, 2023 **(irrelevant)** 17. Newcastle travel guide – February 1, 2024 **(travel)** 18. ‘Hays Travel missold me my holiday. Can I get a refund?’ – February 9, 2024 **(travel)** 19. 16. We’re experts in Zurich — these are the sights and experiences not to miss – February 10, 2024 **(travel)** 20. 9 best places to visit in Malta for your next Mediterranean break – February 15, 2024 **(travel)** 21. Never mind 10,000 steps: 5,000 a day could hold back disease – March 11, 2024 **(irrelevant)** |

## Appendix 16: Novara Media Results

This table illustrates all *Novara Media* search results

| **Novara Media** | |
| --- | --- |
| Search terms: “15-minute city”/ “15-minute cities” | |
| **Pre-Pandemic** | **Post Pandemic** |
| **Included** | **Included** |
| N/A | 1. This is How Britain Can Actually Prepare for Extreme Weather – July 26, 2022 2. How Conspiracy Theorists Made Air Pollution the Latest Front in the Culture War – March 15, 2023 |
| **Excluded** | **Excluded** |
| N/A | 1. Meet the Climate Activists Who Moved Cross-Country to Build a New Civilisation – October 6, 2023 (irrelevant) |
| Search Term: “20-minute neighbourhood”/ “20-minute neighbourhoods” | |
| **Pre-Pandemic** | **Post-Pandemic** |
| **Included** | **Included** |
| N/A | N/A |
| **Excluded** | **Excluded** |
| N/A | N/A |
| Search Term: “Walkable city”/ “Walkable cities” | |
| **Pre-Pandemic** | **Post-Pandemic** |
| **Included** | **Included** |
| N/A | N/A |
| **Excluded** | **Excluded** |
| N/A | N/A |

## Appendix 17: Spiked Magazine Results

This table illustrates all *Spiked Magazine* search results

| **Spiked Magazine** | |
| --- | --- |
| Search terms: “15-minute city”/ “15-minute cities” | |
| **Pre-Pandemic** | **Post Pandemic** |
| **Included** | **Included** |
| N/A | 1. The madness of the ‘15-minute city’ - October 25, 2022 2. The ‘15-minute city’ is not a conspiracy theory – January 22, 2023 3. It’s true – the climate fanatics are coming for your car – February 23, 2023 4. The classist war on the car – May 10, 2023 5. It’s not a conspiracy theory – there really is a war on the car – August 30, 2023 6. The daddy state – December 22, 2023 7. A year of green gaslighting – December 25, 2023 |
| **Excluded** | **Excluded** |
| N/A | 1. Our best articles of 2022 – December 31, 2022 **(irrelevant)** 2. The truth about the BBC’s war on ‘disinformation - May 23, 2023 **(irrelevant)** 3. Greta and the green war on the working class – June 20, 2023 **(irrelevant)** 4. The classist lunacy of Net Zero – June 26, 2023 **(irrelevant)** 5. No, Sadiq Khan’s ULEZ is not going to ‘save lives’ - August 6, 2023 **(irrelevant)** |
| Search Term: “20-minute neighbourhood”/ “20-minute neighbourhoods” | |
| **Pre-Pandemic** | **Post-Pandemic** |
| **Included** | **Included** |
| N/A |  |
| **Excluded** | **Excluded** |
| N/A |  |
| Search Term: “Walkable city”/ “Walkable cities” | |
| **Pre-Pandemic** | **Post-Pandemic** |
| **Included** | **Included** |
| N/A |  |
| **Excluded** | **Excluded** |
| N/A |  |

## Appendix 18: List of Included Articles, Hyperlinks, Publication, Stance on 20MNs, and Potential Funding Conflicts of Interest

This table illustrates all included articles by publication with hyperlinks, stance on 20MNs, and potential funding conflicts of interest noted.

|  | **Article Name & Hyperlink** | **Date Published** | **Publication** | **Political Leaning** | **Stance** | **Potential Funding from Fossil Fuel Industry?** |
| --- | --- | --- | --- | --- | --- | --- |
|  | [The Pandemic Emptied Europe’s Cities. What Will Bring People Back?](https://www.nytimes.com/2021/02/11/world/europe/workers-europe-cities-pandemic.html) | February 11, 2021 | NYT | Mainstream (left-centre) | Supportive | Not found |
|  | [How One San-Francisco Street Survived the Pandemic](https://www.nytimes.com/2021/10/11/us/clement-street-san-francisco-pandemic.html) | October 11, 2021 | NYT | Mainstream (left-centre) | Supportive | Not found |
|  | [The 15-Minute City: Where Urban Planning Concepts Meets Conspiracy Theories](https://www.nytimes.com/2023/03/01/world/europe/15-minute-city-conspiracy.html) | March 1, 2023 | NYT | Mainstream (left-centre) | Supportive/Critical of 20MN conspiracy theories | Not found |
|  | [City Life, Culture Wars and Conspiracy Theories](https://www.nytimes.com/2023/03/06/opinion/city-walkability-culture-wars-conspiracy-theories.html) | March 6, 2023 | NYT | Mainstream (left-centre) | Supportive/Critical of 20MN conspiracy theories | Not found |
|  | [He Wanted to Unclog Cities, Now He’s ‘Public Enemy No. 1’](https://www.nytimes.com/2023/03/28/technology/carlos-moreno-15-minute-cities-conspiracy-theories.html) | March 28, 2023 | NYT | Mainstream (left-centre) | Supportive/Critical of 20MN conspiracy theories | Not found |
|  | [Right Wing Panic Over ‘15-Minute Cities’ About Freedom for Cars, Not People](https://www.counterpunch.org/2023/02/24/right-wing-panic-over-15-minute-cities-about-freedom-for-cars-not-people/) | February 24, 2023 | Counterpunch | Far-left | Supportive/Critical of 20MN conspiracy theories | Not found |
|  | [To Address The Loneliness Epidemic, The Feds Want To Control Your Town And Friends](https://thefederalist.com/2023/05/30/to-address-the-loneliness-epidemic-the-feds-want-to-control-your-town-and-friends/) | May 30, 2023 | The Federalist | Far-right | Against | Yes – via anonymous donations made through Donor’s Trust (33) |
|  | [Anti-Air Travel Climate Psychos Want To Abolish Freedom Of Movement](https://thefederalist.com/2023/10/06/anti-air-travel-climate-psychos-want-to-abolish-freedom-of-movement/) | October 6, 2023 | The Federalist | Far-right | Against | Yes – via anonymous donations made through Donor’s Trust (33) |
|  | [Waltham Forest, the suburb that pioneered the ‘20‑minute neighbourhood'](https://www.thetimes.com/article/waltham-forest-the-suburb-that-pioneered-the-20-minute-neighbourhood-fm0dkw6bs#:~:text=Motorists%20were%20furious%20when%20a,the%20experiment%20is%20a%20success&text=The%20birth%20of%20one,greeted%20by%20a%20noisy%20wake) | September 20, 2020 | TOL | Mainstream (right-centre) | Supportive | Not found |
|  | [What are 15-minute cities and why are antivaxxers so angry about them?](https://www.thetimes.com/article/what-are-15-minute-cities-uk-oxford-fines-2dvqbdc56) | February 22, 2023 | TOL | Mainstream (right-centre) | Critical of 20MN conspiracy theories | Not found |
|  | [Is the 15-minute city the future of urban living?](https://www.thetimes.com/article/is-the-15-minute-city-the-future-of-urban-living-jl27gmr39?id=21336839644&medium=cpc&gad_source=1) | February 26, 2023 | TOL | Mainstream (right-centre) | Mixed | Not found |
|  | [Scottish minister accused of pushing 15-minute cities conspiracy theory](https://www.thetimes.com/article/scottish-minister-accused-of-pushing-15-minute-cities-conspiracy-theory-29snkn6bt) | October 3, 2023 | TOL | Mainstream (right-centre) | Critical of 20MN conspiracy theories | Not found |
|  | [The new Tory conspiracy theory? A 15-minute stroll is ‘sinister’](https://www.thetimes.com/article/caitlin-moran-15-minute-cities-tory-conspiracy-theory-v5m8f8zpb) | October 28, 2023 | TOL | Mainstream (right-centre) | Supportive/Critical of 20MN conspiracy theories | Not found |
|  | [Meet Carlos Moreno, the man who ignited the car culture wars](https://www.thetimes.com/life-style/article/carlos-moreno-interview-culture-cars-15-minute-cities-0cjwsdzpz) | February 29, 2024 | TOL | Mainstream (right-centre) | Supportive/Critical of 20MN conspiracy theories | Not found |
|  | [Oxford planners drop “toxic” 15-minute city phrase](https://www.thetimes.com/uk/article/oxford-city-council-15-minute-city-phrase-dropped-p8sfrfqzh#:~:text=%E2%80%9CWe%20have%20removed%20the%20phrase,to%20judge%20all%20planning%20applications.) | March 6, 2024 | TOL | Mainstream (right-centre) | Supportive/Critical of 20MN conspiracy theories | Not found |
|  | [Antivax newspaper claims to print 150,000 copies a month](https://www.thetimes.com/article/antivax-newspaper-s-conspiracy-theories-could-lead-to-violent-protests-w0gr6wx6v) | June 16th, 2023 | TOL | Mainstream (right-centre) | Critical of 20MN conspiracy theories | Not found |
|  | [Dunbar developers’ ‘Eco plans’ are just greenwashing](https://www.thetimes.com/article/dunbar-developer-eco-plans-greenwashing-scotland-x39dh90gp) | October 23, 2021 | TOL | Mainstream (right-centre) | Sceptical/Mixed | Not found |
|  | [The best 20-minute neighbourhoods in Scotland](https://www.thetimes.com/article/the-best-20-minute-neighbourhoods-in-scotland-6cdp25f2r#:~:text=Bruntsfield%20in%20Edinburgh%2C%20Midstocket%20in,individual%20health%20status%20was%20highest) | December 4, 2022 | TOL | Mainstream (right-centre) | Supportive but highlights evidence-based concerns | Not found |
|  | [Paris braces for a ‘big bang’ if Anne Hidalgo wins as mayor again](https://www.thetimes.com/article/paris-braces-for-a-big-bang-if-anne-hidalgo-wins-as-mayor-again-s7c2gnrt7) | June 28, 2020 | TOL | Mainstream (right-centre) | Supportive but highlights motorists’ concerns | Not found |
|  | [This is How Britain Can Actually Prepare for Extreme Weather](https://novaramedia.com/2022/07/26/this-is-how-britain-can-actually-prepare-for-extreme-weather/) | July 26, 2022 | Novara Media | Far-left | Supportive | Not found |
|  | [How Conspiracy Theorists Made Air Pollution the Latest Front in the Culture War](https://novaramedia.com/2023/03/15/how-conspiracy-theorists-made-air-pollution-the-latest-front-in-the-culture-war/) | March 15, 2023 | Novara Media | Far-left | Supportive/Critical of 20MN conspiracy theories | Not found |
|  | [The madness of the ‘15-minute city’](https://www.spiked-online.com/2022/10/25/the-madness-of-the-15-minute-city/) | October 25, 2022 | Spiked Magazine | Far-right | Against | Yes – Charles Koch foundation (29,30) |
|  | [The ‘15-minute city’ is not a conspiracy theory](https://www.spiked-online.com/2023/01/22/the-15-minute-city-is-not-a-conspiracy-theory/) | January 22, 2023 | Spiked Magazine | Far-right | Against | Yes – Charles Koch foundation (29,30) |
|  | [It’s true – the climate fanatics are coming for your car](https://www.spiked-online.com/2023/02/23/its-true-the-climate-fanatics-are-coming-for-your-car/) | February 23, 2023 | Spiked Magazine | Far-right | Against | Yes – Charles Koch foundation (29,30) |
|  | [The classist war on the car](https://www.spiked-online.com/2023/05/10/the-classist-war-on-the-car/) | May 10, 2023 | Spiked Magazine | Far-right | Against | Yes – Charles Koch foundation (29,30) |
|  | [It’s not a conspiracy theory – there really is a war on the car](https://www.spiked-online.com/2023/08/30/its-not-a-conspiracy-theory-there-really-is-a-war-on-the-car/) | August 30, 2023 | Spiked Magazine | Far-right | Against | Yes – Charles Koch foundation (29,30) |
|  | [The daddy state](https://www.spiked-online.com/2023/12/22/the-daddy-state/) | December 22, 2023 | Spiked Magazine | Far-right | Against | Yes – Charles Koch foundation (29,30) |
|  | [A year of green gaslighting](https://www.spiked-online.com/2023/12/25/a-year-of-green-gaslighting/) | December 25, 2023 | Spiked Magazine | Far-right | Against | Yes – Charles Koch foundation (29,30) |

# References:

1. United Nations Climate Change [Internet]. [cited 2025 Jun 20]. The Paris Agreement | UNFCCC. Available from: https://unfccc.int/process-and-meetings/the-paris-agreement

2. Bogoch II, Watts A, Thomas-Bachli A, Huber C, Kraemer MUG, Khan K. Pneumonia of unknown aetiology in Wuhan, China: potential for international spread via commercial air travel. J Travel Med. 2020 Jan 14;27(2):taaa008.

3. World Health Organization. World Health Organization. [cited 2025 Jun 20]. Novel Coronavirus – China. Available from: https://www.who.int/emergencies/disease-outbreak-news/item/2020-DON233

4. Willsher K. Paris mayor unveils ‘15-minute city’ plan in re-election campaign. The Guardian [Internet]. 2020 Feb 7 [cited 2025 Jun 20]; Available from: https://www.theguardian.com/world/2020/feb/07/paris-mayor-unveils-15-minute-city-plan-in-re-election-campaign

5. World Health Organization. WHO Director-General’s opening remarks at the media briefing on COVID-19 [Internet]. 2020. Available from: https://www.who.int/director-general/speeches/detail/who-director-general-s-opening-remarks-at-the-media-briefing-on-covid-19---11-march-2020

6. World Economic Forum. World Economic Forum. 2020 [cited 2025 Jun 20]. Now is the time for a ‘great reset’. Available from: https://www.weforum.org/stories/2020/06/now-is-the-time-for-a-great-reset/

7. Reid C. Anne Hidalgo Reelected As Mayor Of Paris Vowing To Remove Cars And Boost Bicycling And Walking. Forbes [Internet]. 2020 Jun 28 [cited 2025 Jun 20]; Available from: https://www.forbes.com/sites/carltonreid/2020/06/28/anne-hidalgo-reelected-as-mayor-of-paris-vowing-to-remove-cars-and-boost-bicycling-and-walking/

8. Carlos Moreno [Internet]. n.d. [cited 2025 Jun 20]. Carlos Moreno - Human scale city - ville pour tous | Accueil. Available from: https://www.moreno-web.net/

9. Dr Jordan B Peterson [@jordanbpeterson]. Twitter (X). 2022 [cited 2025 Jun 20]. Available from: https://x.com/jordanbpeterson/status/1609255646993457153

10. Fletcher N. Nick Fletcher. 2023 [cited 2025 Jun 20]. Nick Fletcher MP | 15 MINUTE CITIES. Available from: https://www.nickfletcher.org.uk/news/nick-fletcher-mp-15-minute-cities

11. Caprotti F, Duarte C, Joss S. The 15-minute city as paranoid urbanism: Ten critical reflections. Cities. 2024;155:10549.

12. World Health Organization. WHO chief declares end to COVID-19 as a global health emergency | UN News [Internet]. 2023 [cited 2025 Jun 20]. Available from: https://news.un.org/en/story/2023/05/1136367

13. Reid C. Rishi Sunak’s Attack On 15-Minute Cities Is ‘Baffling’ And ‘Concerning’ Says Originator Of Concept. Forbes [Internet]. 2023 Oct 2 [cited 2025 Jun 20]; Available from: https://www.forbes.com/sites/carltonreid/2023/10/02/rishi-sunaks-attack-on-15-minute-cities-is-baffling-and-concerning-says-originator-of--concept/

14. Vaughan A. Oxford Planners Drop “toxic” 15-minute city phrase. The Times & The Sunday Times [Internet]. 2024 Mar 6; Available from: https://www.thetimes.com/uk/article/oxford-city-council-15-minute-city-phrase-dropped-p8sfrfqzh#:~:text=%E2%80%9CWe%20have%20removed%20the%20phrase,to%20judge%20all%20planning%20applications.

15. Media Bias/Fact Check [Internet]. 2024. The Times UK and the Sunday times - bias and credibility. Available from: https://mediabiasfactcheck.com/the-times-of-london/

16. Ye J, Skiena S. Mediarank: Computational ranking of online news sources. In: Proceedings of the 25th ACM SIGKDD International Conference on Knowledge Discovery & Data Mining. 2019. p. 2469–77.

17. Media Bias/Fact Check [Internet]. 2024. Novara Media - bias and credibility. Available from: https://mediabiasfactcheck.com/novara-media-bias/

18. Novara Media. Novara Media. n.d. About. Available from: https://novaramedia.com/about/

19. Media Bias/Fact Check [Internet]. 2023. Spiked magazine - bias and credibility. Available from: https://mediabiasfactcheck.com/spiked-magazine/

20. Spiked Magazine. spiked. n.d. About spiked. spiked. Available from: https://www.spiked-online.com/about-spiked/

21. Media Bias/Fact Check [Internet]. 2024. New York Times - bias and credibility. Available from: https://mediabiasfactcheck.com/new-york-times/

22. Media Bias/Fact Check [Internet]. 2024. Counterpunch - bias and credibility. Available from: https://mediabiasfactcheck.com/counterpunch/

23. CounterPunch. About [Internet]. n.d. Available from: https://www.counterpunch.org/about/

24. Media Bias/Fact Check [Internet]. 2024. The Federalist - bias and credibility. Available from: https://mediabiasfactcheck.com/the-federalist/

25. Prewitt A. A dangerous plan to address COVID-19 started with a Portland dermatologist. Willamette Week [Internet]. 2020 Mar 26; Available from: https://www.wweek.com/health-wellness/2020/03/26/a-dangerous-plan-for-addressing-covid-19-started-with-a-portland-dermatologist/

26. Novara Media. Novara Media. n.d. How we’re funded. Available from: https://novaramedia.com/about/how-were-funded/

27. Rosa-Luxemburg-Stiftung. Rosa-Luxemburg Foundation. n.d. About us. Available from: https://www.rosalux.de/en/foundation/about-us

28. Slater T. Green cranks are going after spiked – again. Spiked Magazine [Internet]. 2024 Jun 12; Available from: https://www.spiked-online.com/2024/06/12/green-cranks-are-going-after-spiked-again/

29. Small M. DeSmog. 2018. Revealed: US oil billionaire Charles Koch funds UK anti-environment spiked network. Available from: https://www.desmog.com/2018/12/04/spiked-lm-dark-money-koch-brothers/

30. Farrell J. Network structure and influence of the climate change counter-movement. Nat Clim Change. 2016;6(4):370–4.

31. The New York Times. The New York Times. n.d. The Times and your Data. Available from: https://www.nytimes.com/privacy#:~:text=It%20is%20important%20to%20note,Times%20ads%20on%20other%20websites.

32. Remnick D. Sulzberger on the battles within and against the New York Times. The New Yorker [Internet]. 2023 Jun 10; Available from: https://www.newyorker.com/culture/the-new-yorker-interview/a-g-sulzberger-on-the-battles-within-and-against-the-new-york-times

33. Kotch A. Who funds the federalist? finally, we know [Internet]. 2020. Available from: https://www.exposedbycmd.org/2020/12/09/who-funds-the-federalist-finally-we-know/

34. Guilfoyle M. Subject Positioning: Gaps and Stability in the Therapeutic Encounter. J Constr Psychol. 2015;29(2):123–40.

35. Rushton S, Williams OD. Frames, Paradigms and Power: Global Health Policy-Making under Neoliberalism. Glob Soc. 2012;26(2):147–67.
